# Snapshots of a light-induced metastable hidden phase driven by the collapse of charge order

**DOI:** 10.1126/sciadv.abp9076

**Published:** 2022-07-22

**Authors:** Frank Y. Gao, Zhuquan Zhang, Zhiyuan Sun, Linda Ye, Yu-Hsiang Cheng, Zi-Jie Liu, Joseph G. Checkelsky, Edoardo Baldini, Keith A. Nelson

**Affiliations:** ^1^ Department of Chemistry, Massachusetts Institute of Technology, Cambridge, MA 02139, USA.; ^2^ Department of Physics, Harvard University, Cambridge, MA 02138, USA.; ^3^ Department of Physics, Massachusetts Institute of Technology, Cambridge, MA 02139, USA.; ^4^ Department of Electrical Engineering and Computer Science, Massachusetts Institute of Technology, Cambridge, MA 02139, USA.; ^5^ Department of Physics, The University of Texas at Austin, Austin, TX 78712, USA.

## Abstract

Nonequilibrium hidden states provide a unique window into thermally inaccessible regimes of strong coupling between microscopic degrees of freedom in quantum materials. Understanding the origin of these states allows the exploration of far-from-equilibrium thermodynamics and the development of optoelectronic devices with on-demand photoresponses. However, mapping the ultrafast formation of a long-lived hidden phase remains a longstanding challenge since the initial state is not recovered rapidly. Here, using state-of-the-art single-shot spectroscopy techniques, we present a direct ultrafast visualization of the photoinduced phase transition to both transient and long-lived hidden states in an electronic crystal, 1*T*-TaS_2_, and demonstrate a commonality in their microscopic pathways, driven by the collapse of charge order. We present a theory of fluctuation-dominated process that helps explain the nature of the metastable state. Our results shed light on the origin of this elusive state and pave the way for the discovery of other exotic phases of matter.

## Teaser

A suite of advanced single-shot spectroscopies is used to track the ultrafast formation of a metastable hidden phase.

## INTRODUCTION

Ultrafast light-matter interactions can trigger a plethora of exotic phenomena in quantum materials (*1*, *2*), such as light-induced superconductivity (*3*–*5*), nonlinear phononic control of lattices (*6*–*8*), and photon-dressed topological phases (*9*, *10*). In addition, the study of photoinduced hidden phases (*11*, *12*), i.e., states inaccessible in equilibrium phase diagrams, has recently emerged as a thriving field of research. While many hidden phases that are induced by laser pulses are short-lived (*8*, *13*–*15*), a few such phases can persist indefinitely under suitable environmental conditions (*11*, *12*, *16*, *17*). For such nonequilibrium metastable phases, salient gaps in our understanding remain. Conventional pump-probe spectroscopy methods, being stroboscopic in nature, can provide profound insights into many phase transitions but are not applicable when the material does not return to its initial state after each pump laser shot. Single-shot time-resolved spectroscopy techniques, on the other hand, can capture, with a single pump laser shot, the real-time dynamical evolution of slowly reversible and irreversible processes (*12*, *18*, *19*). Single-shot measurements thereby can offer unique mechanistic insights into the genesis of metastable hidden phases.

**Fig. 1.**
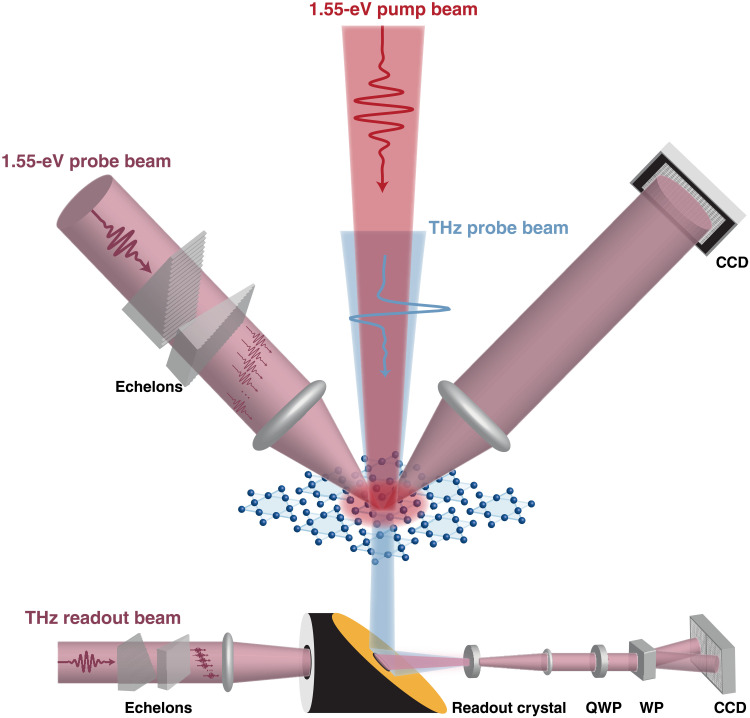
Schematic illustration of dual-echelon single-shot optical reflectivity and THz transmission spectroscopy. The sample is photoexcited by a 1.55-eV pump laser pulse. In single-shot reflectivity, the 1.55-eV probe beam (incident from top left) is passed through a set of dual 20-step echelons and split into a 20 by 20 grid of 400 pulselets with different time delays. These probe pulselets are focused onto the 1*T*-TaS_2_ sample along with the 1.55-eV pump pulse (red). The reflected probe pulselets are detected on different regions of a charge-coupled device (CCD) camera. In single-shot THz transmission measurements, a THz beam is incident on the sample (from above), and the transmitted THz field is focused into an electro-optic readout crystal in which field-induced birefringence gives rise to time-dependent optical polarization rotation proportional to the instantaneous THz field amplitude. A 1.55-eV readout beam (incident from bottom left) is passed through the echelons to generate 400 pulselets that overlap with the THz field in the electro-optic (EO) crystal at different times. The transmitted pulselets are passed through a quarter-waveplate (QWP) and a Wollaston prism (WP), producing two 20 by 20 grids of beams for balanced detection at a CCD camera. In both cases, the resulting grid images are binned and unfolded to obtain a 9.3-ps time-domain trace of transient reflectivity or THz signal all in a single laser shot.

A metastable hidden phase whose origin and formation pathway are highly debated (*11*, *20*, *21*) occurs in the prototype quasi–two-dimensional charge-density wave (CDW) crystal, 1*T-*TaS_2_. This system undergoes successive first-order phase transitions upon cooling. First, from the high-temperature incommensurate CDW state (IC state), 1*T*-TaS_2_ enters a nearly commensurate CDW state (NC state) at 350 K. It then undergoes a phase transition to a commensurate state (C state) at 180 K, below which a Mott gap develops (*22*, *23*). In the C state, an ultrafast photoinduced phase transition to a metastable hidden state (H state) induced by a single laser pulse has been observed at low temperature (<10 K), accompanied by a drop of several orders of magnitude in the resistance (*11*). The highly conductive state can be erased by increasing the sample temperature or annealing thermally with a train of stretched pulses but is otherwise persistent. Because of its intriguing properties and the potential memory applications of this type of photoinduced phase, the metastable H state of 1*T*-TaS_2_ has been investigated by transport measurements (*24*, *25*), stroboscopic pump-probe spectroscopy (*11*, *26*), scanning tunneling microscopy (STM) (*26*, *27*), transmission electron microscopy (*28*), and x-ray diffraction (*21*). However, all the measurements of this persistent phase examine observables characterized before and after switching and thereby cannot provide insights into the initial dynamics of H state formation. In contrast, at higher temperatures (e.g., >70 K), it is argued that the metastable H state can also be induced but only transiently as the system reverts to the pristine C state between shots. Using a three-pulse pump-probe technique at such sample temperatures, a first pulse of sufficient fluence was used to induce a phonon frequency shift characteristic of the H phase that was observed by variably delayed pump-probe measurements (*26*, *29*). Since the metastable H state formed at low temperature is persistent and the one formed at higher temperatures is transient, it remains an open question whether these states are essentially the same and how the system is driven into the H state. Addressing these questions has been a longstanding challenge due to the lack of appropriate tools for tracking the ultrafast formation of long-lived hidden phases. Here, by conducting dual-echelon single-shot time-resolved spectroscopy experiments in both the near-infrared (NIR) and terahertz (THz) spectral ranges (Fig. 1) (*30*), we directly capture the light-induced nucleation, stabilization, conductivity, and relaxation associated with the transformation into the transient or persistent phase and unveil the fundamental connections between the photoinduced states and the pathways into them. Our results provide the first direct measurement of the photoinduced dynamical melting of nanoscale charge order followed by the dynamical formation of a different nanoscale charge ordering, all observed in real time.

## RESULTS

### THz conductivity of the H state

**Fig. 2.**
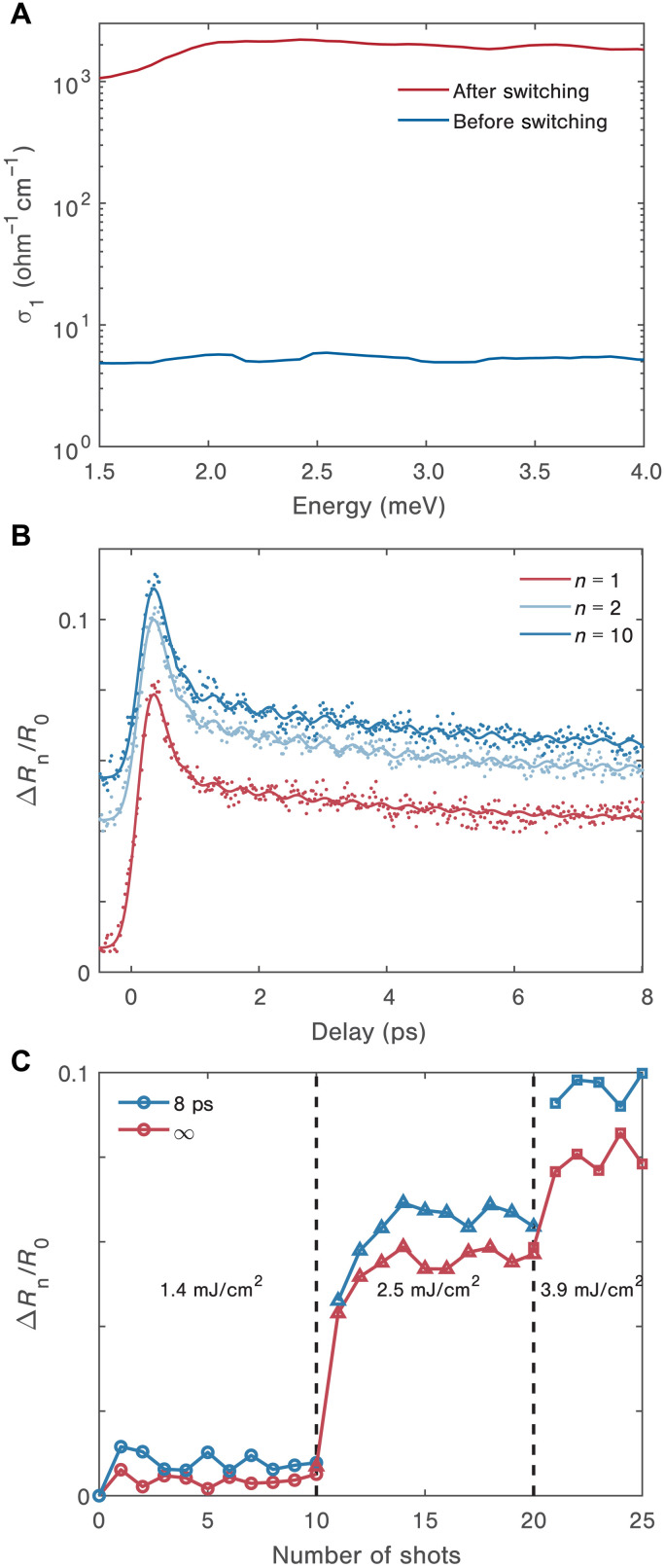
Dynamics of the emergent hidden phase formed after photoexcitation in 1*T*-TaS_2_. (**A**) Steady-state real part of the THz conductivity increases by two orders of magnitude following switching. (**B**) Shot-by-shot transient reflectivity measurements (dots) at 7.8 K following irradiation with 2.5 mJ/cm^2^ 1.55-eV pump pulses are shown along with the corresponding fits to biexponential decays and oscillatory responses (solid lines). Ten shots were recorded at this fluence but only the 1st, 2nd, and 10th are reproduced here for clarity. Each irradiation was separated by more than 10 s. (**C**) The corresponding shot-to-shot change in steady-state reflectivity (red) and 8-ps reflectivity (blue) over the full sequence of irradiations. Note that *n* = 1 in (B) corresponds to the 11th shot in (C).

We first establish the creation of the H phase in 1*T*-TaS_2_ upon photoexcitation with a single, intense laser pulse at 1.55 eV (fluence *F* = 2.5 mJ/cm^2^). To this aim, we measure the THz optical conductivity in the steady state before and after excitation at 7.8 K. Before irradiation (blue curve in Fig. 2A), the value of the real part σ_1_ (∼5 ohm^−1^ cm^−1^) and its featureless spectrum are consistent with the presence of a fully gapped insulating state (*31*). Pumping the material results in a drastic change of the steady-state conductivity (red curve in Fig. 2A): The conductivity reaches values of 10^3^ ohm^−1^ cm^−1^, which is about an order of magnitude larger than its counterpart in the NC CDW phase at 230 K (*31*). This more conducting behavior is one of the fingerprints of the metastable H phase and is consistent with previous transport measurements in the DC limit (*24*, *25*). Unlike DC transport, THz spectroscopy has access to the conductivity over a wide spectral range, providing insights into the nature of the charge carriers. We observe that the value of σ_1_ is relatively energy independent above 2 meV and slightly suppressed below. This response is not captured by the conventional Drude model, suggesting a suppression of long-range transport due to electronic or structural disorder (*32*).

### Single-shot transient reflectivity of the persistent H state

With the formation of the H state established, we seek to address key questions about its underlying dynamics. The first unknown regards the time scale for H state nucleation. To measure this, we use single-shot transient reflectivity, with both pump and probe pulses centered at 1.55 eV. Figure 2B shows the data acquired at 7.8 K upon sample irradiation with 1 (red), 2 (light blue), and 10 (blue) consecutive pump pulses (keeping for each one the same incident fluence used in the THz experiment of Fig. 2A). Upon absorption of the first pump pulse, we observe a time-resolution limited increase in the sample reflectivity, followed by a subpicosecond relaxation and a slower decay over a few picoseconds (red dots). The relaxation dynamics are well captured by a biexponential fitting function, with relaxation times of 0.17 and 3.7 ps, as shown by the red solid curve. For long time delays (>8 ps), the slow decay converges to a large (∼5%) and stable reflectivity offset that never recovers back to the original, pretime-zero value. This new value of the NIR reflectivity provides a distinct signature of H state formation, this time at photon energies as large as a few electron volts. We also observe that the process of H state writing/erasing is completely reversible: Cycling the sample temperature between 7.8 and 80 K erases the H state and restores the pristine C state (see fig. S2). Upon cooling down to 7.8 K, the H state can be created again by applying another laser pulse at 2.5 mJ/cm^2^ fluence, resulting in a transient reflectivity curve identical to the red trace in Fig. 2B. After the first shot, an additional shot at the same 2.5 mJ/cm^2^ fluence induced a further small change in the static reflectivity, as shown in Fig. 2C (shots 11 and 12; the first 10 shots are at 1.4 mJ/cm^2^ fluence). Subsequent shots at 2.5 mJ/cm^2^ fluence (shots 13 to 20) resulted in no further systematic change. See fig. S3 for the entire sequence. The increased static reflectivity value after the first shot differs only slightly from its value at 8-ps time delay after photoexcitation, and there is only a modest change after 1-ps delay. From this, we infer that the stabilization of the H state is complete within several picoseconds of the first pump laser shot.

Having revealed the ultrafast dynamics of H state formation triggered by a single laser pulse, we obtain a broader overview of the switching behavior and evolution of the H state through single-shot measurements at several excitation fluence regimes. Figure 2C shows a sequence of 25 single-shot irradiations at three different fluences and, for each shot, compares the reflectivity change at 8 ps with the steady-state reflectivity of the sample long after the shot. We observe that irradiating the sample with one pump pulse at 1.4 mJ/cm^2^ produces only a slight increase (maximum of 1%) in the sample reflectivity at 8 ps and that this reflectivity signal decays over time. Further irradiation with a train of nine pulses at the same fluence does not result in substantial changes in the signal behavior. As discussed above, when the pump fluence exceeds a threshold value of *F*_th,7.8K_ = 2.5 mJ/cm^2^, the sample undergoes a sudden increase in steady-state reflectivity. Another sudden change occurs when the fluence is increased to 3.9 mJ/cm^2^, an observation that is consistent with the photoinduced metallicity (*28*). At this fluence, we see once again that the full extent of switching is induced by the first shot, and further shots at the same fluence have no further systematic effects. As in the 2.5 mJ/cm^2^ fluence case, the steady-state reflectivity value reached after the first each shot differs by only a modest amount from the value at 8 ps (or 1 ps) delay following excitation, which indicates that switching at this fluence also occurs on a picosecond time scale. Note that the switching observed here is entirely reversible upon thermal cycling (see note S2), which rules out any formation of the amorphous (or A) state known to persist up to room temperature (*26*, *33*). We also note that at all fluences, including the first shots at the two high fluences, the relaxation kinetics show a subpicosecond decay component followed by a slower multipicosecond decay (the values are indicated in table S1). The decay rates at 1.4 and 2.5 mJ/cm^2^ fluences are similar, although no switching occurs at the lower fluence. At the highest fluence, the fast decay component is faster, and the slow component is slower but the same trends seen at lower fluence hold (see fig. S3C). From these results, we attribute the first process to electronic relaxation due to typical electron-phonon interactions while the following processes are dominated by thermal fluctuations that involves the formation of disordered structures (see the “Theory of pump-induced dynamics” section). The consequences of these charge-order fluctuations are entirely different from the static thermal response that should result in a NIR reflectivity change of opposite sign to what is observed, i.e., a decrease in ∆*R/R*.

We also gain insights into the pathway through which the system enters the H state (*11*, *29*) by observing the CDW order parameter. The single-shot traces in Fig. 2B and fig. S3 show 2.4-THz coherent oscillations in the time domain, which are characteristic signatures of the CDW amplitude mode (*34*–*37*). The amplitude of this collective mode becomes weaker as more of the sample switches into the H state (first shots at 2.5 and 3.9 mJ/cm^2^ fluences), indicating that the melting of the original CDW order is tied to the formation of the metastable state (see note S2 and fig. S4).

### Averaged single-shot transient reflectivity of the transient H state

To resolve the dynamical behavior of the photoinduced phase transformation without any persistent response, we raise the sample temperature to 80 K and conduct single-shot NIR transient reflectivity measurements. In this regime, we demonstrate that the lifetime of the H state is shortened to less than 20 ms. The recovery is too slow to allow conventional pump-probe measurements at our 1-kHz laser repetition rate, but we can track the CDW dynamics with high signal-to-noise ratio by averaging over many single-shot traces. We find that the threshold fluence for H state formation is reduced to *F*_th,80K_ ∼ 0.55 mJ/cm^2^, i.e., a value that is notably lower than *F*_th,7.8K_. This suggests that the ground state is more susceptible to disruption from photoexcitation at the higher temperature compared to when the system is deeply trapped in the C state at the lower temperature.

**Fig. 3.**
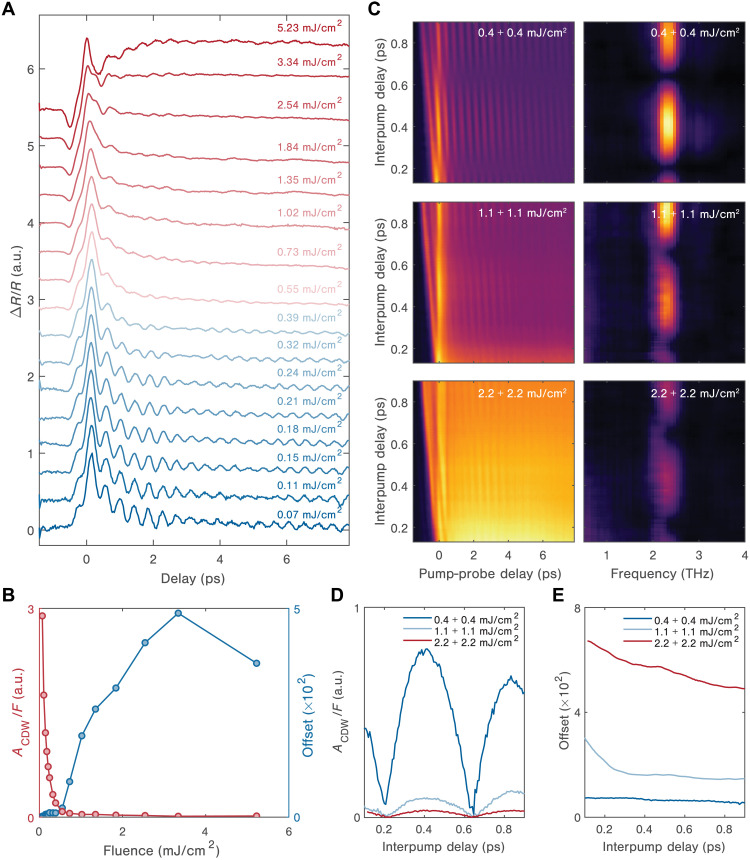
Far-from-equilibrium NIR transient reflectivity of 1*T*-TaS_2_ at 80 K. (**A**) Single-shot transient reflectivity measurements averaged over 100 shots at 80 K following photoexcitation with an 1.55-eV pump pulse over a wide range of excitation fluences. (**B**) The CDW amplitude mode strength normalized by the fluence (red) and the offset response (blue) extracted from the fits to (A) as a function of excitation fluence. At low fluence, the offset response is nearly zero and only appears above a critical threshold fluence of 0.55 mJ/cm^2^, above which it rapidly increases with increasing fluence. For the CDW amplitude mode, the coherent response is strongest at low fluence and then drops steadily as the fluence is increased, nearly disappearing at the same threshold fluence. (**C**) Single-shot transient reflectivity measurements as a function of both interpump delays and pump-probe delays with three distinct fluence regimes: 0.4 + 0.4 mJ/cm^2^ or low fluence regime, 1.1 + 1.1 mJ/cm^2^ or medium fluence regime, and 2.2 + 2.2 mJ/cm^2^ or high fluence regime. (**D**) The amplitude mode strength and (**E**) offset extracted from the fits to (C) for the three fluence regimes as a function of interpump delay. a.u., arbitrary units.

Figure 3A shows the measured single-shot spectroscopy data at various incident fluences, with each curve obtained from averaging 100 single-shot traces. Unlike in our conventional pump-probe measurements—in which sample damage occurs above 1.5 mJ/cm^2^ (see note S3)—single-shot experiments enable us to enter a hitherto unexplored excitation regime (up to 5.23 mJ/cm^2^) and provide a comprehensive view of the H state formation. Below the threshold fluence for the creation of the H state (*F < F*_th,80K_), we observe that the sinusoidal signal due to the amplitude mode appears promptly after the initial electronic response and oscillates with a frequency of 2.4 THz. Increasing the incident fluence results in the weakening of the amplitude mode, indicating a transient suppression of the CDW order parameter (*34*, *38*). Figure 3B shows the strength of the amplitude mode, *A*_CDW_, and the *t >* 8 ps offset signal component as a function of fluence (fitting procedures are described in note S5 and fits are shown in fig. S8). Once the fluence exceeds *F*_th,80K_, the mode intensity reduces almost to zero, signaling a nearly complete melting of the CDW order. Simultaneously, an offset emerges, a response that resembles the dynamics involved in entering the persistent H state at 7.8 K. This unique feature is not observed in the conventional pump-probe data in either this or previously reported works (*26*, *29*) and is not compatible with a mere increase in the lattice temperature (see fig. S5 and note S4). Therefore, we can conclude that the offset is the fingerprint of the metastable H state at 80 K. Above *F*_th,80K_, the offset increases monotonically with the excitation fluence, up to 3.3 mJ/cm^2^, before being suppressed at the highest fluence. Here, the time-domain trace also changes markedly, with the signal upturning after the initial impulsive response and with a new oscillation emerging at a frequency of 2.1 THz. We ascribe the latter to the *E_g_* phonon mode (*35*, *39*). Unlike the amplitude mode, the *E_g_* phonon mode is not directly coupled to the instantaneous perturbation of the charge density but rather to the periodic lattice distortion (*35*). In this framework, the disappearance of the CDW amplitude mode and the persistence of the *E_g_* phonon mode at high fluence suggest that the melting of the CDW does not fully suppress the periodic lattice distortion (see note S5 and figs. S7 and S8) (*40*, *41*).

From this dataset, we can also establish that the CDW melting leading to H state creation proceeds nonthermally. At *F*_th,80K_, the lattice temperature remains well below the equilibrium CDW melting temperature (see note S4) (*42*). We further verify this by performing a series of double–pump pulse single-shot reflectivity experiments, varying the interpump delay while keeping other parameters unchanged (Fig. 3C, left column). For nonthermal melting, one expects an increase in the offset and a further suppression of the amplitude mode oscillation as the interpump delay decreases, since the system is effectively pumped by a single stronger pulse before it fully relaxes. However, for thermal melting, no such variation would occur at short interpump delays (within several picoseconds) since the total energy deposited by the pump pulse pair does not change substantially. We explore three different excitation regimes for the pump pulses: low (*F* < *F*_th,80K_), medium (*F* > *F*_th,80K_), and high (*F* > > *F*_th,80K_) fluence. We then perform a Fourier transform analysis of the oscillatory signal along the pump-probe delay axis (Fig. 3C, right column). At all fluences, we observe that the amplitude mode is modulated as the interpump delay is changed, manifesting itself as interference effects in the Fourier transform signal. However, as the fluence increases, the amplitude mode oscillations become less prominent. The offset response exhibits a strong dependence on interpump delay. The strengths of the amplitude mode and the offset for these three regimes are extracted from the Fourier transforms and the fits as functions of interpump delay (Fig. 3, D and E). In the regime with medium fluence, the offset drops substantially when the interpump delay is increased, accompanied by the recovery of the amplitude mode strength after the second to third periods of delay. In the high fluence regime, the amplitude mode is further suppressed, and the long-lived offset becomes prominent, reflecting that the material enters the photoinduced metastable H state. These observations reaffirm that the melting is nonthermal, showing a strong dependence on the peak carrier density, and that a threshold density of initially absorbed photons is required to trigger the long-lived metastable state.

### Single shot NIR pump THz probe signals

**Fig. 4.**
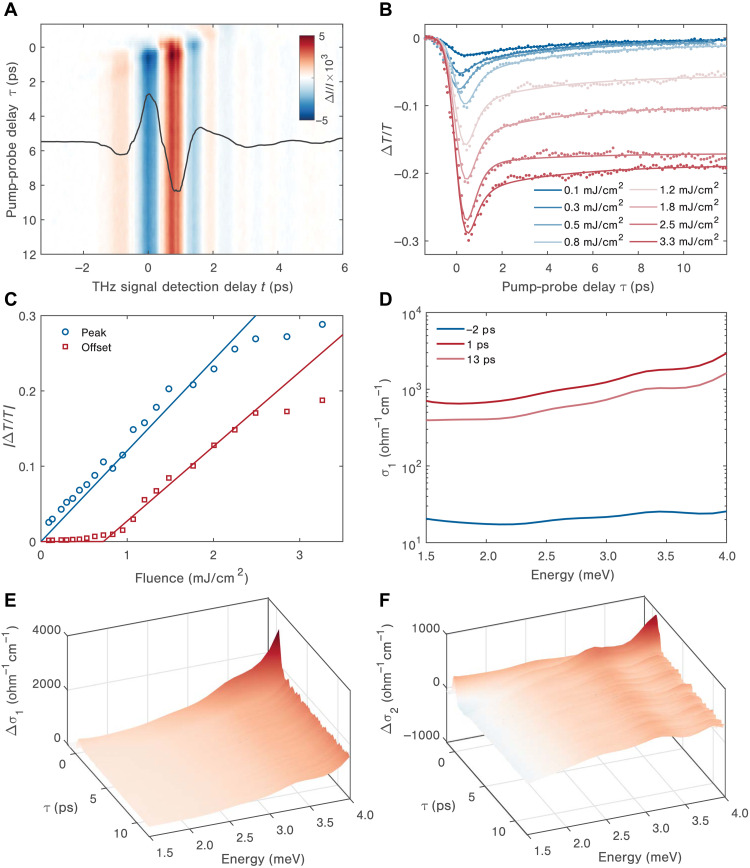
Far-from-equilibrium THz conductivity of 1*T*-TaS_2_ at 80 K. (**A**) Photoinduced change in the THz electric field trace, *E*(*t*), at 80 K as a function of pump-probe delay, τ, with 1.55-eV pump and an excitation fluence of 3.3 mJ/cm^2^. The transmitted THz field profile without photoexcitation (black line) is sketched with the same horizontal time axis. In the data shown, the pump pulse decreases the transmission of THz light through the sample, with the effect persisting for the entire range of delay times following the pump pulse. (**B**) Spectrally integrated differential transmission of the main THz peak through the sample following photoexcitation over a large range of excitation fluences. (**C**) Peak response (blue) and offset response (red) extracted from the fits to (B) as a function of excitation fluences. The offset response only shows up above a critical excitation fluence and increases with fluence above the threshold. The solid lines show roughly linear trends in the peak response and the above threshold offset with respect to the excitation fluence up to about 2 mJ/cm^2^ fluence. (**D**) Comparison between the real part of the transient THz conductivity before and after photoexcitation of 3.3 mJ/cm^2^. There is an increase of two orders of magnitude in the transient conductivity following photoexcitation. (**E** and **F**) The spectro-temporal evolution following the photoinduced change with fluence of 3.3 mJ/cm^2^ [same as (A)] in the real and imaginary parts of the THz conductivity.

Last, we establish how the initial CDW state transforms into the H state by investigating the pump-induced changes to the THz conductivity with single-shot THz spectroscopy, a recently developed technique (*19*, *43*) for measuring long-lived low-energy responses (details are given in Materials and Methods). The THz trace is read out by 400 electro-optic sampling probe pulses that arrive at the sample at different delay times relative to the pump laser pulse, allowing us to collect the full time-dependent conductivity response on each shot. In the present study, we repeated the measurements at a repetition rate of 50 Hz, which still allows a high total data acquisition rate given the number of time points collected on each shot. This also ensures that the observed dynamics arise from the effect of each individual pump pulse rather than any cumulative effects of multiple pulses (no variation in signal was seen at still lower repetition rates). Figure 4A shows the photoinduced change in the transmitted THz field, *E*(*t*), at 80 K as a function of pump-probe delay, τ, under the excitation fluence of 3.3 mJ/cm^2^. By analyzing the change in the spectrally integrated THz field transmission (∫|*E*(ω)|*d*ω), we can extract the reduced THz transmission after photoexcitation, presented in Fig. 4B for a large range of excitation fluences. Also shown in solid lines are fits to biexponential decays convoluted with the instrument response function. Although the time resolution is limited by the THz pulse duration, we observe a response that is reminiscent of the NIR transient reflectivity traces, exhibiting similar time scales and changing considerably above a critical excitation threshold around *F*_th,80K_. At larger fluences, a long-lived offset dominates after the initial resolution-limited response and the following relaxation process. We then plot the offset and peak transmissivity retrieved from the fits in Fig. 4B. As shown in Fig. 4C, the peak amplitude displays an approximately linear scaling as a function of excitation fluence (especially in the low fluence regime), while the offset signal shows an increasing trend only above *F*_th,80K_. Figure 4 (E and F) displays the spectro-temporal evolution of the real (∆σ_1_) and imaginary (∆σ_2_) parts of the differential THz conductivity following excitation with a single 3.3 mJ/cm^2^ pump pulse. Upon laser excitation, ∆σ_1_ is increased by two orders of magnitude (Fig. 4E), and ∆σ_2_ becomes negative at low energies. For longer pump-probe delay, both ∆σ_1_ and ∆σ_2_ are compatible with a Drude-Smith type of response, similar to the steady-state conductivity when the H state is switched on at 7.8 K. This response is typical of confined carriers affected by strong backscattering. In this case, scattering likely occurs at either the boundaries of the metallic domains of the H state or the boundary between the photo-switched H state and the pristine C state, which results from the limited penetration depth of the 1.55-eV pump beam.

### Theory of pump-induced dynamics

Previous quantitative models of the H state are based on a picture of electron-hole asymmetry that yields an ordered structure of polaron clusters (*11*). It was posited that due to an asymmetry of the band structure, the photoexcited electrons and holes relax at different rates, resulting in excess charges in the delocalized bands. These excess charges then annihilate with polarons creating voids that will aggregate and stabilize upon reaching sufficient density. Quantitatively, the temporal evolution of the system was modeled after the relaxation dynamics of three different reservoirs of carriers coupled to the lattice. Choosing a minimal representation for the free energy and chemical potential, the emergence of a stable point corresponding to a phase with excess voids was found at high electron temperature, which was ascribed to the photoinduced H state. Although we believe that this explanation remains valuable, it does not consider the possibility that interacting CDW layers could affect the metallicity, as suggested by more recent works (*20*, *21*, *44*, *45*). To account for these observations both in and out of equilibrium, we propose a theory that does not require the introduction of a new order parameter on top of the existing CDW order. Instead, the formation of the H state can be described by the mechanism of fluctuation explosion (*46*) that follows the collapse of charge order. We note that thermal fluctuations have been found to play important roles in the pump-induced dynamics of other CDW systems as well (*47*–*51*).

The pump-induced dynamics of the CDW order can be described by the space and time-dependent order parameter field ψ(**r***,t*) = ψ_0_(*t*) + δψ(**r***,t*) on each two-dimensional layer of 1*T*-TaS_2_. The free energy is in the usual Landau form

$$F/E_{c}=-\alpha \psi^{2}+\xi_{0}^{2}{\left(\nabla \psi \right)}^{2}+\psi^{4}$$ (1)

where *E*_c_ is the condensation energy density, ξ_0_ is the bare coherence length, and α(*T*) is *O*(1) at zero temperature *T*. Since there are 13 different stable free-energy minima corresponding to aligning an arbitrary lattice site with the 13 different tantalum atoms in the star of David, the order parameter cannot be represented by a simple real number. However, the discrete symmetry means that its scaling behavior can be equivalently described by the Ising order parameter with two minima in Eq. 1.

To describe the dynamics induced by a strong pump above the switching threshold, we assume that the order parameter fields evolve according to relaxational [or “model A” (*52*)] dynamics

$$\frac{1}{\gamma }\partial_{t}\psi (r,t)=-\frac{1}{E_{c}}\frac{\delta F(t)}{\delta \psi (r,t)}+\eta (r,t)$$ (2)

where γ = 1*/*τ is the intrinsic relaxation rate. We make the natural assumption that the applied NIR pump field raises the temperature of the electronic system such that α(*T*(*t*)) and thus *F*(*t*) are time dependent. η is the random noise satisfying the fluctuation-dissipation theorem $\langle \eta_{i}(r,t)\eta_{i}(r^{\prime },t^{\prime })\rangle =\frac{2T(t)}{\gamma_{i}E_{c}}\delta (r-r^{\prime },t-t^{\prime })$.

**Fig. 5.**
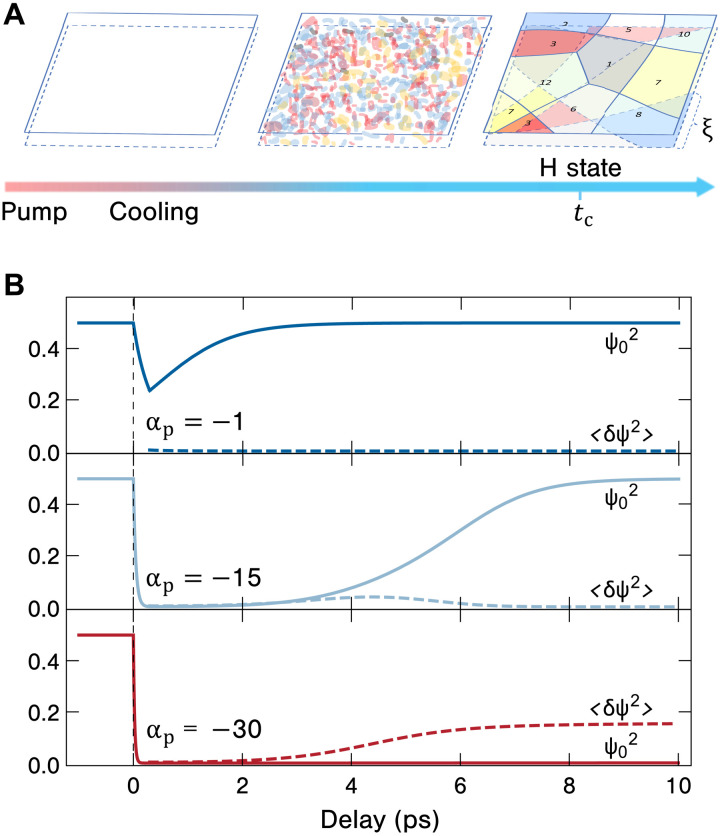
Theory of fluctuation-dominated mechanism. (**A**) Schematic illustration of the pump-induced dynamics of the sample. The horizontal arrow is the arrow of time following the pump pulse. The two planes represent two adjacent 1*T*-TaS_2_ layers. Each number labels a domain in the H state corresponding to 1 of the 13 possible low-energy CDW states. (**B**) The time evolution of the square of the mean field order parameter and its fluctuations computed from model A (Eq. 2). The effect of the pump is modeled as a time-dependent quadratic term coefficient α(*t*) = α_0_ + (α_p_ − α_0_)[Θ(*t*) − Θ(*t* − *t*_p_)] where α_0_ = 1 is its static value. The pump time is chosen as *t*_p_ = 0.3 ps since the pump duration is 0.1 ps, while the electronic system needs approximately 0.2 ps to cool down. The Ginzburg number and the intrinsic CDW relaxation time are the same as Eq. 4. Three different values of α_p_ (−1, −15, and −30) are chosen to represent three different pump fluence regimes (low, medium, and high).

In this framework, our results can be explained by Fig. 5A and the bottom of Fig. 5B, following the spirit of (*46*). A strong pump pulse transiently heats up the electronic system and renders α negative such that the mean field CDW order parameter ψ_0_ decays to a negligible value, even smaller than its thermal fluctuations. After the pump, the electron system quickly cools down by transferring its excess energy to the lattice within a time scale of 0.3 ps. Then, the CDW fluctuations δψ(**r***,t*) grow exponentially, forming random domains (*46*) within each layer. Since the interlayer coupling is much weaker than the intralayer one, we treat each layer individually such that the random domains of adjacent layers are not correlated. Given any two overlapping domains on adjacent layers, there is very little chance that they are perfectly aligned to form the original insulating C state [center of the David star on top of each other (*20*, *21*)]. Therefore, one has an H state of random domains, which is mostly metallic due to this altered stacking order (*20*, *21*, *44*, *45*). As shown in Fig. 5B, low (e.g., α_p_ = −1) and medium (e.g., α_p_ = −15) fluence pump pulses partially quench the mean field CDW order $\psi_{0}^{2}$. In the subsequent cooling process, $\psi_{0}^{2}$ fully recovers, consistent with the low fluence regime of our experiments. At high fluences (e.g., α_p_ = −30), $\psi_{0}^{2}$ is quenched to vanishingly small values such that in the cooling process, the exponentially growing thermal fluctuations 〈δψ^2^〉 dominate, which results in a long-lived disordered metastable state, i.e., H state, that forms within roughly 6 ps. Successive irradiations at the same fluence would repeat the same cooling process and thereby contribute no further changes to the degree of H state formation, while further irradiations with higher fluences will result in more thermal fluctuations and thereby increase the amount of photoinduced metallicity, in agreement with our experimental observations in Fig. 2B.

In principle, there are driving forces to relax this H state back to the C state: The weak interlayer coupling provides a force to shift the local CDW horizontally to align the overlapping domains, and the intralayer dynamics of Eq. 2 tend to shorten the total domain wall length to reduce the energy. These relaxation mechanisms account for the transience of the H state discovered at high temperature; however, there are several reasons why this metastable H state is long-lived or even persistent at low temperature. First, because of the frustration of domain overlap on adjacent layers, the intralayer restoring force tends to cancel out. Second, the domain walls may be pinned by disorder, which provides barriers for their motion and enhances the lifetime of the H state exponentially as the temperature is lowered.

From a theoretical perspective, our single-shot measurements also provide a benchmark that enables us to extract several fundamental parameters of the Landau-Ginzburg-Wilson theory for the system. In the fast-cooling limit (electron-phonon scattering time shorter than the order parameter growth time *t*_c_), the time needed to form the metastable H state and the domain size are (*46*, *53*)

$$t_{c}\sim \frac{\tau }{4\alpha }\text{ln}\frac{1}{\zeta },\;\xi \sim \xi_{0}\sqrt{2/\alpha }\sqrt{\text{ln}\frac{1}{\zeta }}$$ (3)

where ζ is the Ginzburg parameter (or “Ginzburg number”) that determines the accuracy of the mean field picture. From the STM images of the H states induced by optical and electrical pulses (*26*, *44*, *54*), the bare coherence length (roughly the width of the domain walls at a temperature much lower than *T*_c_) is about ξ_0_ = 3 nm, while the domain size is about ξ = 10 nm. Together with the new observation that the evolution time is *t*_c_ = 3.7 ps, we obtain the Ginzburg parameter and the intrinsic relaxation time of the in-plane CDW

$$\zeta ∼4\times 10^{-3},\;\tau ∼2.7\text{ps}.$$ (4)

The small value of ζ means that the theory used here is reasonable.

## DISCUSSION

Together, our single-shot time-resolved measurements combined with the stochastic time-dependent Ginzburg-Landau theory establish a fundamental connection between the transient and persistent H states in 1*T*-TaS_2_. Their similarities in dynamics after photoexcitation show that H state formation follows the same pathway, regardless of the transient or persistent nature of the resulting phase. In both regimes, we observe direct evidence for the ultrafast nature of photoinduced metallicity. Upon above-threshold laser excitation, the CDW order melts on a nonthermal time scale faster than our experimental resolution. After the collapse of the electronic order, instead of returning to the original C state, thermal fluctuations (*46*) dominate the following relaxation process, allowing the metastable H state to set in on a time scale of several picoseconds. Such a fast time scale characterizing the stabilization of the H state is in stark contrast to the time scale involved in other photoinduced persistent insulator-to-metal transitions, for example, when the formation of the conductive states relies on slowly developing ferromagnetic domains (*19*). Our findings support a picture in which the ultrafast collapse of a charge gap can lead to a lasting change in electronic structure and modification of the free-energy landscape, as confirmed by theory. In this case, the primary difference between the two temperature regimes is a kinetic one, with the eventual relaxation of the H state and the reformation of the C state being faster at high temperature. Note that our findings prompt comparison with previous results that have inferred the time scale of H state formation at much higher temperatures (e.g., >80 K) with a three-pulse stroboscopic technique (*26*, *29*). In those measurements, the correspondence of this transient state with the persistent one was unclear, and no comparison of their photoinduced dynamics was available. Direct tracking of single-shot switching events without introducing additional pump pulses clarifies the formation of the H state under conditions directly comparable with previous reports of the photoinduced metallicity in 1*T*-TaS_2_ (*11*, *28*) and yields crucial information not accessible otherwise. It is hoped that future single-shot measurements on monolayer and few-layer 1*T*-TaS_2_ will unambiguously demonstrate the role of fluctuations to stabilize the H state. Furthermore, our observations show distinct optical responses for the formation of the H state (i.e., the concomitant increases in NIR reflectivity and THz conductivity) that demonstrate that the H state is substantially different from the NC state and, in general, from any other equilibrium states. These results highlight the potential to use the functionality of H states on-demand and over a wide range of time scales, providing additional control for future optoelectronic devices based on ultrafast photoresponses. More fundamentally, our single-shot techniques can be used to study other materials with photoinduced metastable phase transitions that remain unexplained, such as systems with multipolar orders (*55*), ferroelectric superlattices (*56*), and molecular solids with light-induced superconductivity (*4*, *57*).

## MATERIALS AND METHODS

### Single-crystal synthesis

Single crystals of 1*T*-TaS_2_ were synthesized via a chemical vapor transport process (*58*). The process we adopted here has been described elsewhere (*59*). We first mixed Ta powder (Alfa Aesar, 99.97%) and S pieces (Sigma-Aldrich, 99.998%) with an off-stoichiometric ratio of 1:2.02; the mixture was placed in an evacuated quartz tube and kept at 970°C for 2 days and subsequently quenched in cold water. A second evacuated quartz tube containing the resulting TaS_2_ along with added I_2_ was placed in a temperature gradient from 920°C (source) to 820°C (sink) for 2 weeks. At the end of the growth, the quartz tube was quenched in water. Powder x-ray diffraction was performed to confirm that the resulting crystals are of the 1*T*-TaS_2_ phase.

### Single-shot NIR transient reflectivity

Single-shot dual echelon NIR transient reflectivity measurements were conducted by using the output of a Ti:Sapphire laser system (800 nm, 1 kHz, 3 mJ, and 70-fs pulses) and down-counting to 10 Hz via a Pockels cell and polarizing beamsplitter. A mechanical shutter synchronized to the laser output was used to select individual laser pulses. Most of the laser power was sent into a modified Michelson interferometer that generated two delayable pump pulses. Approximately 100 μJ of the laser output was split off and passed through dual echelons, resulting in a temporally offset 20 by 20 grid of pulselets spanning about 9.3 ps. These pulselets were then focused onto the sample along with the pump pulse (spot size ∼ 600 μm) in a transient reflectivity geometry that included a reference grid of beamlets (not shown in Fig. 1) for normalization of the signals as described previously (*30*). A 4f imaging systems was used to relay the signal and reference grids onto a charge-coupled device (CCD; Hamamatsu ORCA-ER). These grids were then combined, binned, and unfolded to generate a pump-probe transient reflectivity trace measured in each laser shot (*18*, *30*). All fluences reported are incident fluences, *F*_abs_, which were calculated by dividing the incident 1.55-eV pump power by the product of the horizontal and vertical beam diameters at full width at half maximum. The latter values were retrieved by fitting the pump beam profile at the sample position to a two-dimensional Gaussian function. Conversions to the absorbed fluence, *F*_abs_, can be performed via

$$F_{\text{abs}}=F_{\text{inc}}(1-R_{1.55\;\text{eV}})$$

where the 1.55-eV reflectivity of 1*T*-TaS2, e.g., at 77 K, *R*_1.55 eV_ = 0.49 (*37*). A fluence of 0.55 mJ/cm^2^ therefore corresponds to an absorbed fluence of ~0.28 mJ/cm^2^.

### Single-shot optical pump THz probe spectroscopy

The laser output was down-counted to 50 Hz, and no sample damage was observed in the measurement. A total of 1 mJ of the laser output was used to generate THz probe pulses via optical rectification in LiNbO_3_ using a tilted pulse front geometry. The remaining ∼1 mJ was divided between optical pump and electro-optic sampling beams. The THz probe and optical pump were focused onto the sample with spot sizes of roughly 1 and 2 mm, respectively. Single-shot readout of the THz waveform was performed by passing the electro-optic sampling beam through dual echelons and then focusing the resulting grid of beamlets and THz onto a 1-mm GaP crystal. These grids were then imaged onto a CCD (Andor Zyla) after being split into perpendicularly polarized images by passing through a quarter waveplate and a Wollaston prism as shown in Fig. 1. The final THz waveform was obtained by binning, unfolding, and then subtracting the two resulting time traces. To measure the two-dimensional optical pump-THz probe maps, the THz probe and optical pump were chopped at 25 and 12.5 Hz, respectively. The single-shot THz readout method has been described in detail previously (*19*, *43*).

The THz conductivity of the photoexcited region was obtained from the experimentally determined optically induced THz transmission spectra, $\tilde{T}(\omega )$, using the Tinkham equation (*60*)

$$\tilde{\sigma }(\omega )=\frac{n_{s}+1}{Z_{0}d}(\frac{1}{\tilde{T}(\omega )+1})$$ (5)

where *n*_s_ is the index of the substrate, *Z*_0_ is the impedance of free space, and *d* is the sample thickness or the optical penetration depth. In the data analysis, we accounted for the penetration depth mismatch between the NIR pump and THz frequencies by treating the optically excited region as its own sample, with thickness commensurate with the optical penetration depth, i.e., *d* = 45 nm. The overall thickness of the sample used in the THz transmission measurements was ∼50 μm.

### Calculation details

The state of the system is, in principle, described by a probability functional ρ[ψ(*r*, *t*)] that satisfies a Fokker-Planck equation (*46*, *52*) equivalent to Eq. 2. To reduce the complexity, one may focus only on the mean field order parameter ψ_0_(*t*) = 〈ψ〉 and the two-point correlation function (the fluctuations) *D*(*r*, *t*) = 〈δψ(0, *t*)δψ(*r*, *t*)〉 where 〈〉 means the expectation value with respect to ρ.

In the high fluence case (Fig. 5A and bottom of Fig. 5B), the mean field order parameter is almost completely quenched by the pump. In the subsequent cooling process, the exponential growth of order parameter fluctuations and the formation of the domain structure are described by equation 17 and section IV of (*46*).

For low and medium fluences, one needs to take into account the competition between the fluctuations *D*(*r*, *t*) and the nonnegligible mean field ψ_0_(*t*). Making the “Hartree Fock” approximation for the probability functional (i.e., that ρ[ψ(*r*, *t*)] = Π_k_ρ_k_(ψ_k_) is a direct product of ρ for each momentum mode), Eq. 2 leads to a set of coupled nonlinear differential equations

1γ∂tψ0=2αψ0−4(ψ02+3D(0,t))ψ0,1γ∂tD(r,t)=4[α−ξ02∇2−6(D(0,t)+ψ02)]D(r,t)+2ζξ0dδ(r) (6)

where *d* = 2 is the space dimension. One may solve Eq. 6 for the pump-induced dynamics, with the initial condition $\psi_{0}=\sqrt{\alpha /2}$ and *D*(*r*, *t*) being its equilibrium value.

A further approximation justified in (*46*) leads to a simpler approach. In the pumping process (0 *< t < t*_p_), *D*(*r*, *t*) ∼ ζ is tiny so that we neglect *D* in the first of Eq. 6. In the cooling process (*t > t*_p_), *D*(*r*, *t*) is well approximated by an exponential form *D*(*r*, *t*) = 〈δψ^2^〉_t_*e*^−*r*^2^/ξ(*t*)^2^^ with the initial condition being $D(r,t_{p})\sim \zeta e^{-r^{2}/\xi_{0}^{2}}$. By neglecting the noise term [this does not substantially change the results as justified in (*46*)], Eq. 6 becomes that for $\sigma_{0}^{2}$, 〈δψ^2^〉, and ξ(*t*)—the numerical solution of which yields Fig. 5B.

## Supplementary Materials

### This PDF file includes:

Notes S1 to S5 Figs. S1 to S9 Tables S1 to S3 References
