# Supplementary material for: Snapshots of a light-induced metastable hidden phase driven by the collapse of charge order
Source: Sci Adv. 2022 Jul 22;8(29):eabp9076. doi: 10.1126/sciadv.abp9076 (PMC9307249; doi:10.1126/sciadv.abp9076)
Supplement: Supplementary file 1 — Notes S1 to S5 Figs. S1 to S9 Tables S1 to S3 References [file sciadv.abp9076_sm.pdf]

Science Advances
AAAS

Supplementary Materials for

Snapshots of a light-induced metastable hidden phase driven by the collapse of charge order

Frank Y. Gao *et al.*

Corresponding author: Keith A. Nelson, kanelson@mit.edu; Edoardo Baldini, edoardo.baldini@austin.utexas.edu

Sci. Adv. **8**, eabp9076 (2022)
DOI: 10.1126/sciadv.abp9076

This PDF file includes:

Notes S1 to S5
Figs. S1 to S9
Tables S1 to S3
References

Supplementary Note 1: Data analysis for static and ultrafast spectrally-resolved THz transmission measurement

To analyze the THz conductivity of the H state we apply the Drude-Smith Model (*32,61,62*), which has been widely used in the analysis of carrier transport dynamics in confined systems (*63-65*). Per the standard formulation, the Drude-Smith conductivity $\tilde{\sigma}(\omega)$ is written as:

$$\tilde{\sigma}(\omega) = \frac{Ne^2\tau/m^*}{1 - i\omega\tau}\left(1 - \frac{c}{1 - i\omega\tau}\right) \tag{1}$$

where N is the carrier density, e is fundamental electron charge and m^* is the effective carrier mass. τ and c are phenomenological constants associated with the model, the Drude-Smith scattering time and the "localization constant", the latter of which ranges from 0 to -1. If $c = 0$, there is no localization and the standard Drude model is recovered. If $c = -1$, we attain the limit of strong localization where all DC conductivity is suppressed. The THz conductivities of the H state at 80 K are shown with fits to the Drude-Smith model in Figure 1. The model does reasonably well in capturing the imaginary part of conductivity over the spectral range, being negative at low photon energies (a hallmark of back-scattering) and then increasing at higher photon energies. However, the real part of the conductivity shows an increase at high frequencies, which the model does not capture. While a possible source of this rise is the tail of an optical phonon resonance at 6.6 meV, attempts to fit a Lorentz oscillator were unsuccessful, which implies that this feature is due to the electronic degrees of freedom. Furthermore, this feature becomes more prominent as the fluence is raised beyond the threshold for H state formation and, therefore, may result from a spectral weight transfer that occurs when the system enters the H state. With regards to the Drude-Smith model parameters, we extract a Drude-Smith scattering time of $\tau = 270$ fs and a localization constant of $c = -0.76$. This τ value, though long relative to Drude scattering times obtained at room temperature, is reasonable due to the reduced electron-phonon scattering at 80 K and is in line with measurements in other systems at the same temperature (*63*). The localization parameter c of -0.76 indicates considerable carrier localization and back-scattering which may be due to (a) the limited penetration depth of the optical pump resulting in a heterogeneity of the H phase formation along the depth of the sample and/or (b) the formation of metallic domains whose boundaries inhibit long-range transport.(*27,28*)

Supplementary Note 2: Single-shot switching to the H state at low temperature

A. Temperature hysteresis after the formation of the H state

In this section, we present and analyze the effect of raising the temperature after H state formation. As in previous studies (*11*), we use conventional stroboscopic pump-probe measurements to track the collective mode signatures in the pristine C state and the H state as we cycle the temperature. Specifically, we measure the NIR transient reflectivity traces with weak pump

fluence ($F = 0.33$ mJ/cm^2) as we slowly cool down the temperature starting from 80 K. When the temperature is stabilized at $\sim$ 10 K, we irradiate the pristine sample with an intense NIR laser pulse ($F = 2.5$ mJ/cm^2, the same fluence used in the single-shot NIR transient reflectivity measurements) to create the H state. After the H state is formed, we then measure the pump-probe responses under the same condition (0.33 mJ/cm^2 fluence) as for the pristine C state. Figure 2b shows the NIR transient reflectivity traces acquired in the pristine C and H states at 11 K. Comparing the traces, the difference before and after irradiation is consistent with previous reports and reaffirms the formation of the H state in our measurements. To show that the H state can be erased upon heating, transient reflectivity traces are also taken as we increase the temperature. At $\sim$ 60 K, the response almost completely reverts to the previous one. To gain a more quantitative understanding of these data, we fit all the traces and plot both the decay rate and the amplitude mode frequency as a function of temperature (Fig. 2c and Fig. 2d). Cooling down the temperature only causes small changes in both parameters, but irradiation with an intense laser pulse at the lowest temperature causes the decay rate to increase significantly and causes the amplitude mode to soften. Upon heating to 60 K and beyond, both parameters can be restored, suggesting the H state is thermally annealed to the C state at this temperature.

To show that the process is reversible, we again cool down the temperature to 7.8 K so that the H state can be created again by applying another NIR laser pulse at 2.5 mJ/cm^2. We then acquire the single-shot transient reflectivity traces for the first 5 shots. Again, the first shot creates a large static reflectivity offset ($\sim$4%) with following shots only incrementing this static response by small amounts. The similarity between these data and the responses shown in Fig. 2a shows the single-shot transient reflectivity response during this stage of H state formation reveals the reversibility of the H state in our measurements. Note that we did not observe the amorphous state (or A state) that can be stable up to room temperature, in line with previous reports that the A state appears depending on sample and measurement conditions.(*26,33*) As such, any discussion about the A state is beyond the scope of our current work.

B. Full sequence of the single-shot transient reflectivity traces

In the main text, we present the shot-to-shot change in static reflectivity and 8 ps reflectivity over a series of single-shot irradiations. Here, we show the full sequence of these single-shot transient reflectivity traces along with fits using the function

$$A_1 e^{-\Gamma_1 t} + A_2 e^{-\Gamma_2 t} + A_{CDW} e^{-\Gamma_{CDW} t} \cos(\omega_0 t + \phi) + C, \tag{2}$$

where A_1, A_2 and A_{CDW} are the amplitudes of the first two decay processes and the perturbation of the CDW order parameter, Γ_1, Γ_2 and Γ_{CDW} are the corresponding relaxation rates, ω_0 and ϕ are the frequency and phase of the amplitude mode oscillations, and C is the offset parameter giving the signal level at long times. (All fit parameter values are shown in Tables 1 and 2.) This fitting function is further convolved with a Gaussian instrument response function (FWHM of 180 fs) to reproduce the traces in solid lines in Fig. 3a-c. Unlike the single-shot transient reflectivity traces in Fig. 2b, in which all transient reflectivity traces are normalized by the pre-switched static reflectivity, here the change of NIR reflectivity in each trace is always

normalized by the new pre-time-zero offset signal after the last shot to give better visualization of the underlying dynamics. From this set of data, we observe an oscillatory responses due to the amplitude mode, with the signal-to-noise ratio limited by the single-shot nature of these measurements. The Fourier spectra corresponding to the background-subtracted oscillatory components are also shown in Fig. 3d-f. We can analyze the strength of the amplitude mode from both the fits and Fourier spectra of these single-shot responses, as displayed in Fig. 4. For the first ten shots with pump fluence at $F = 1.4$ mJ/cm^2, the amount of switching to the H state is negligible so we do not observe any shot-to-shot variation of the amplitude of the collective mode oscillation. At higher fluences however, we observe nontrivial behavior of the amplitude mode when the C state switches to the H state. The amplitude mode is suppressed at $F = 2.5$ mJ/cm^2 and $F = 3.9$ mJ/cm^2 for all traces, but most significantly for the first shots at these fluences. As determined by the static reflectivity in Fig. 2c in the main text, these two irradiations are also identified as the major events where significant amount of switching to the H state occurs. This observation is reminiscent of the behavior at 80 K while the H state is still transient, where the suppression of the amplitude mode is simultaneously accompanied by a long-lived offset that signals the appearance of the H state. By combining these factors, we conclude that the formation of the H state involves the melting of the CDW order along with the redistribution of the quasiparticle energy.

Supplementary Note 3: Conventional pump-probe results

To compare the single-shot technique to conventional pump-probe measurements, we acquire a series of fluence-dependent conventional transient reflectivity measurements under the same sample conditions as the optical single-shot measurements conducted at 80 K. For these measurements, the laser repetition rate is set at 1 kHz and the pump is chopped at 500 Hz. The resulting time traces are shown in Fig. 5a. By extending the pump-probe delays to more than 30 ps, we can identify collective mode behaviors at low fluences through the analysis of the oscillatory components, as shown in Fig. 5b. At the lowest fluence ($F = 0.21$ mJ/cm^2), the oscillatory response is dominated by the underdamped amplitude mode, persisting even after 30 ps, which is further reflected as a sharp peak at 2.4 THz in the Fourier transform (see Fig. 5c). In addition to the amplitude mode, other conventional phonons are also present in the Fourier transforms, with their frequencies at 2.1 THz, 3.2 THz and 3.9 THz. At higher fluences, these phonon modes are slightly suppressed, but the most prominent change is the weakening and broadening of the amplitude mode, as observed in our single-shot data. However, as we further increase the fluence to above 0.6 mJ/cm^2, we notice a significant difference between conventional and single-shot results. Whereas at the higher fluences, there is no visible offset in the conventional pump-probe measurements, in the single-shot data, the offset becomes significant. Furthermore, by raising the fluence beyond 1.5 mJ/cm^2 at what is still a low fluence in the context of our single-shot measurements, we observe a continuous drop in signal that suggests photo-induced sample damage. This observation is also supported by previous results, which show that the system does not fully recover between shots (i.e. < 1 ms) at 80 K when the pump fluence exceeds 1 mJ/cm^2.(*26*) This indicates that the heating effect from successive laser pulses can accumulate over time. In summary, collecting conventional pump-probe data

without artifacts is challenging when working with slowly-relaxing systems, such as $1T$-TaS_2 (i.e. ¿ 1 ms), as it is often difficult to determine that the data do not reflect the intrinsic material dynamics. We suggest that single-shot time-resolved spectroscopy is an ideal tool to study these systems with long-lived responses.

Supplementary Note 4: Nonthermal phase transition

In the main text, we indicate that our two-pump experiments exclude an interpretation of the long-lived offset signals at 80 K as thermally excited states. In this section, we explain in detail why this alternative scenario can be ruled out.

A. Estimation of the CDW melting in a thermal process

First, we estimate the minimum excitation fluence required to raise the lattice temperature due to a thermal process, i.e. a simple transfer of the laser energy to the lattice, from 80 K to the CDW melting temperature ($T_{IC} = 350$ K). Assuming quasi thermal equilibrium, this fluence is equal to

$$F = \frac{1}{d(1-R)} \int_{80K}^{T_{IC}} dT\, C(T), \tag{3}$$

where R is the static reflectivity ($\sim$ 0.49)(*37*), and d is the penetration depth ($\sim$45 nm)(*31*) of 1.55 eV excitation photons at 80K. The heat capacity $C(T)$ is calculated using the Debye model:

$$C(T) = 9N_A k_B \left(\frac{T}{\Theta_D}\right)^3 \int_0^{\Theta_D/T} dx \frac{x^4 e^x}{(e^x-1)^2}, \tag{4}$$

where the experimentally determined Debye temperature, $\Theta_D = 237$ K, was taken from a previous calorimetry measurement(*66*). The value of $C(T)$ ranges from 13 J mol^{-1}K^{-1} at 80 K to 24 J mol^{-1}K^{-1} at 350 K. From the above calculation, the minimal excitation fluence required to collapse the charge order is about $F = 1.7$ mJ/cm^2, which is well above the threshold fluence for the offset to appear and the amplitude mode to disappear. Note that in our estimate, we do not consider any effects of non-equilibrium electron scattering or transport, nor do we consider heat diffusion from the initially heated region of the lattice itself, and we thereby underestimate the required fluence. Hence, we show that the melting of the CDW and the creation of the H state must be a non-thermal process.

B. Temperature dependence of the optical properties

Next, we emphasize that the optical properties of the light-induced metastable state at 80 K are in stark contrast with those of any state near thermal equilibrium. Starting at 77 K, raising the temperature results in a decrease in the optical reflectivity at 1.55 eV, as evidenced by previous reports (*31,67*). However, the photoinduced H state at 80 K bears the signature of a long-lived

positive reflectivity offset, which rules out the observed metastable state as a thermally induced state.

To further investigate the differences between photoinduced thermal and nonthermal effects, we perform the single-shot transient reflectivity measurements on the same sample at 220 K while warming up. See Fig. 6. At this temperature, the sample enters the NC state and we observe a positive initial reflectivity response followed by a negative $\Delta R/R$, which results from an increase in lattice temperature. If $1T$-TaS_2 in the C state were photo-thermalized into the NC state, one would expect $\Delta R/R$ to flip sign at longer delays. To confirm this, we further increase the fluence used in the single-shot transient reflectivity measurements at 80 K. We note that only when we increase the fluence up to $F = 7$ mJ/cm^2 , do we observe a similar negative response. This negative transient along with the absence of the positive offset indicates that the thermal phase transition only plays a key role in such a high fluence regime. This further confirms that the photoinduced long-lived metastable state is non-thermal in nature.

Supplementary Note 5: Analysis of collective modes observed in single-shot NIR transient reflectivity at 80 K

A. Collective mode oscillations under single pump excitation

The behavior of the collective mode oscillation at 80 K, as seen in Fig. 3 of the main text, helps provide additional clues about the transition to the H phase. The extracted oscillatory signals and spectra from the fluence-dependent NIR transient reflectivity experiments are shown in Fig. 7. The corresponding time-domain oscillatory and decay signals are shown in Fig. 8 along with fits to the function,

$$A_e e^{-\Gamma_e t} + A_{CDW} e^{-\Gamma_{CDW} t} \cos(\omega_0 t + \phi) + C, \tag{5}$$

with the parameters given in Table 3. Here, A_e and A_{CDW} are amplitudes of the electronic response and the fluctuation of the CDW order parameter, Γ_e and Γ_{CDW} are the corresponding relaxation rates, ω_0 and ϕ are the frequency and phase of the amplitude mode oscillations, and C is the offset parameter giving the signal level at long times. At low fluences, we observe a beating pattern in the oscillation which is likely due to interference among multiple phonon modes of similar frequencies, in line with previous observations (*35*). Spectrally, the individual peaks cannot be resolved and instead show up as a broad peak at 2.4 THz which matches that of the A_{1g} CDW amplitude mode. As the fluence is increased, the amplitude mode weakens and nearly disappears at about 0.55 mJ/cm^2, which also coincides with the melting of the CDW order and the first appearance of the H phase. Simultaneously, the main oscillation frequency blue-shifts slightly. As the fluence is increased even further and the main mode continues to weaken, an additional mode at 2.1 THz appears at 1.35 mJ/cm^2 and then grows to eventually dominate the signal at the highest fluence. The divergence in strength and behavior between these two modes indicates different excitation pathways. Whereas the main oscillation is strong at low fluences, being triggered via displacive excitation (*31,37,68*), and weakens with the melting of the CDW, the additional mode increases with fluence in the intermediate-fluence regime and only becomes prominent as the CDW amplitude mode weakens. As mentioned in the main

text, the 2.1 THz mode matches the known E_g mode of $1T$-TaS_2 (*35,39*) which is excited via impulsive stimulated Raman scattering (*35,69*). Furthermore, this mode is associated with the periodic lattice distortion of the material so its prominence at the high fluences here suggests that the distortion persists even when the CDW has been thoroughly melted.(*40*)

B. Double pump measurements and the 2.1 THz mode

To isolate the 2.1 THz oscillation, we conduct a series of double pump experiments as a function of fluence where the inter-pump delay is set to approximately cancel the main 2.4 THz CDW amplitude mode ($\tau = 0.64$ ps). The resulting oscillatory spectra and the strengths of the 2.1 THz mode as a function of pump fluence are shown in Fig. 9. For these measurements, we note that because of the nonthermal nature of the melting of the CDW and the relatively large inter-pump spacing used here, the degree of CDW melting due to two pumps of a given fluence is much more comparable to the response to a single pump at that fluence as opposed to a single pump with double the fluence. At the lowest fluences, we observe that the 2.1 THz mode is weak and difficult to resolve due to the limited signal-to-noise ratio of the single-shot scans. As the fluence increases, the 2.1 THz mode strength increases roughly linearly before plateauing and decreasing at the highest fluences. For intermediate fluences, both the 2.1 and 2.4 THz modes coexist, confirming they are distinct modes with different origins. Since the amplitude mode is tied to the CDW order, its strength weakens in this fluence regime along with the melting of the charge order. However, the E_g mode is not as sensitive to the CDW order as the A_{1g} mode so its strength continues to increase with increasing fluences. Furthermore, the 2.1 THz mode is weak at lower fluences but is much clearer here, supporting the notion that this mode does exist across a wide range of fluences but is normally masked by the strong A_{1g} CDW oscillation. These results support the assignment of the 2.1 THz mode to the previously observed E_g mode.

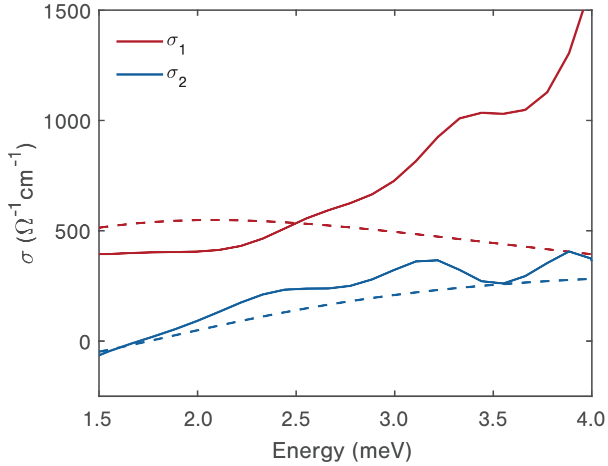

Fig. 1. Drude-Smith model of H state THz conductivities. The real (solid red) and imaginary (solid blue) THz conductivities of the H state at 80 K (τ =13 ps, F = 3.3 mJ/cm^2) are shown along with the corresponding fits to the Drude-Smith conductivity model (dashed) with $\tau = 270$ fs and $c = -0.76$.

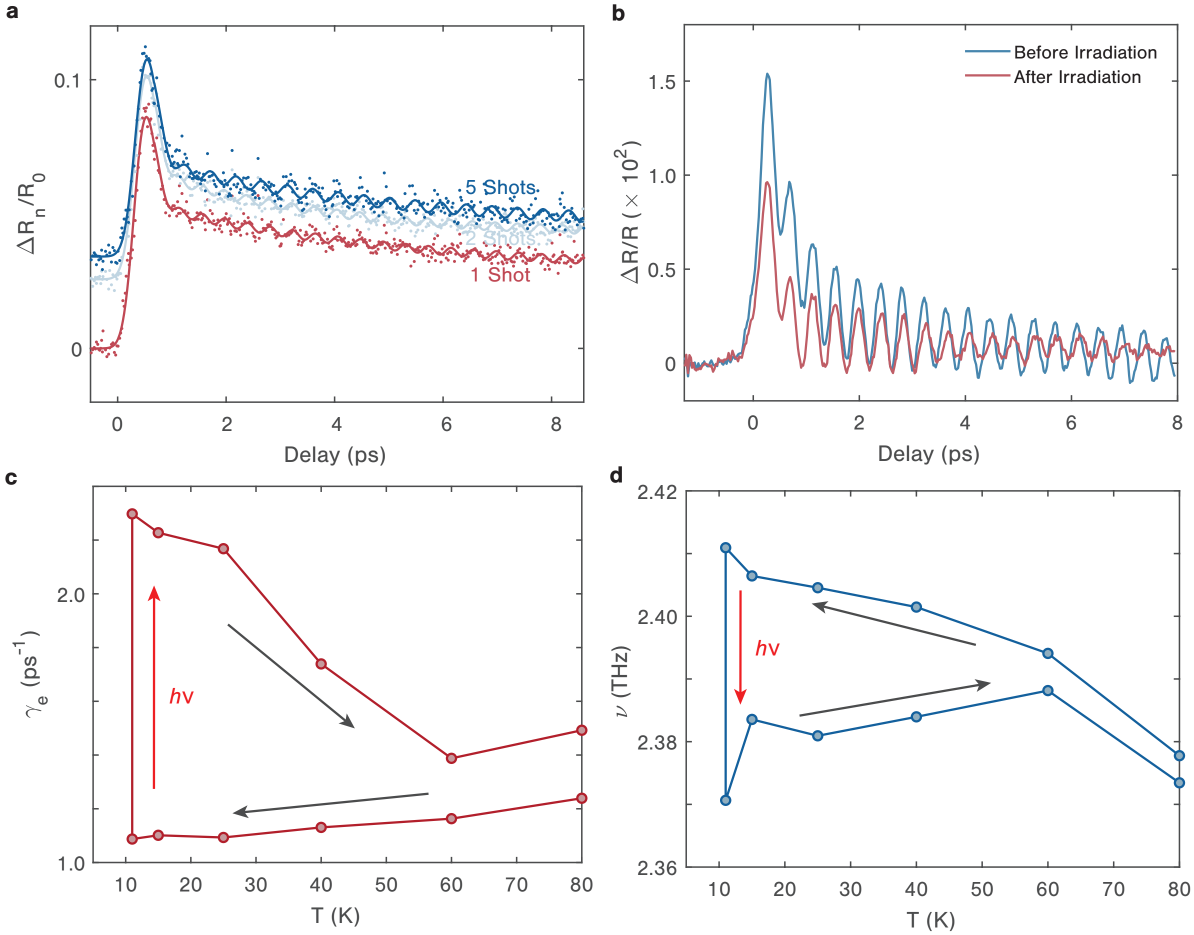

Fig. 2. Creation and relaxation of the H state via temperature cycling. a, A shot-to-shot sequence on the same spot as Fig. 2b pumped at 2.5 mJ/cm^2 after temperature cycling from 7.8 K to 80 K and back to 7.8 K shows identical switching behavior. **b**, Pump-probe transient reflectivity with weak (0.33 mJ/cm^2) pump excitation before (blue) and after (red) the creation of the H state at 11 K. We collected the pump-probe data while cooling from 80 K to 11 K before switching and then while warming back up to 80 K after switching and fit both **c**, the initial decay and **d**, the collective mode frequency for all traces.

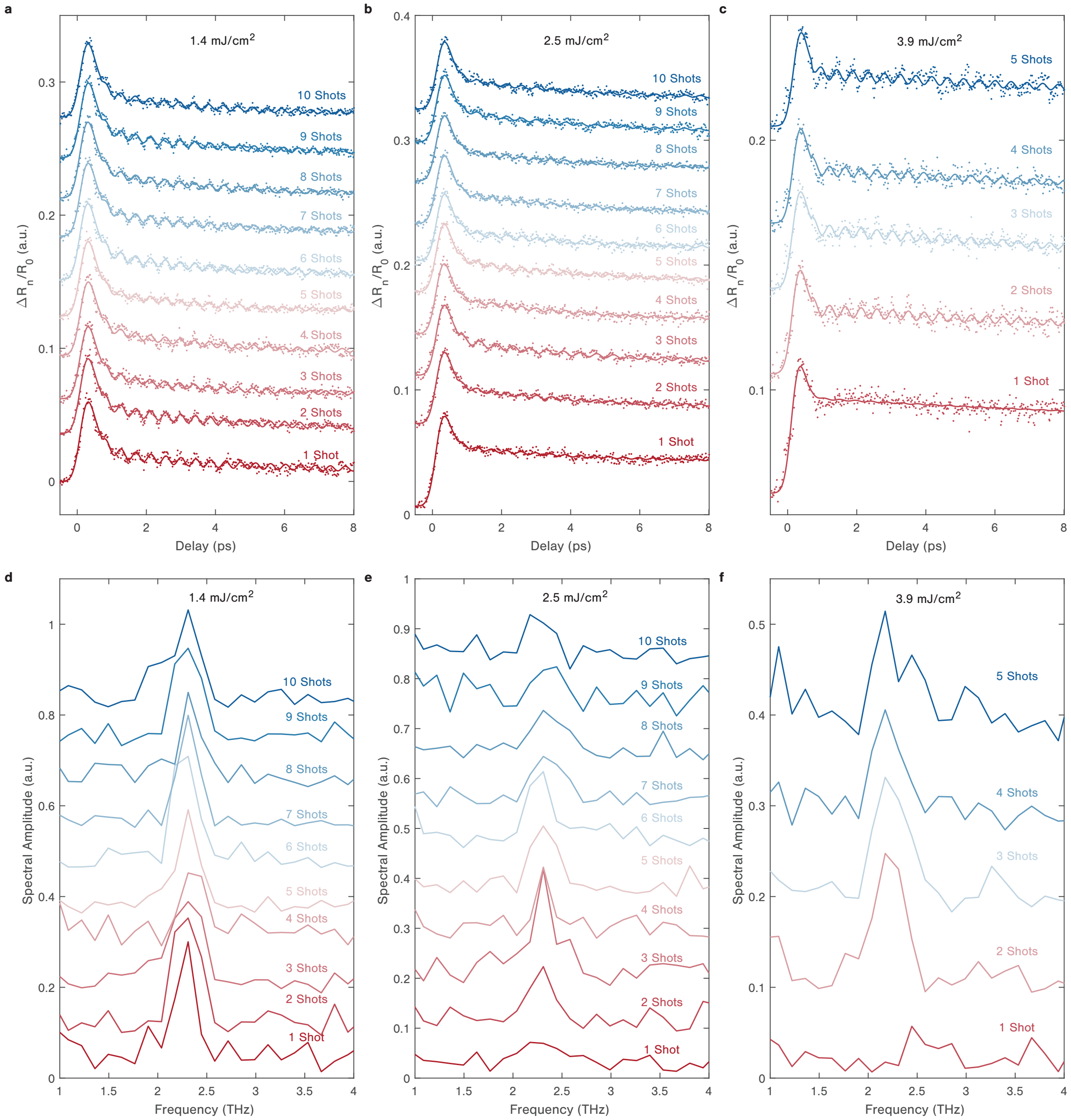

Fig. 3. The entire sequence of the single-shot reflectivity measurements at 7.8 K for Figure 2b. a, The first ten shots conducted with an incident fluence of 1.4 mJ/cm^2. **b**, Ten shots with an incident fluence of 2.5 mJ/cm^2 following the irradiation in **a**. **c**, 5 shots with an incident fluence of 3.9 mJ/cm^2 following the irradiation in **b**. The solid lines are fits to a biexponential decay and damped oscillation convoluted with the instrument response function. In (a)-(c) the sweeps have been shifted vertically for clarity. The absolute shifts in reflectivity from shot to shot are shown in Fig. 2c. **d**, **e**, **f**, Fourier transforms corresponding to the background-subtracted oscillatory signals in **a**, **b**, **c**.

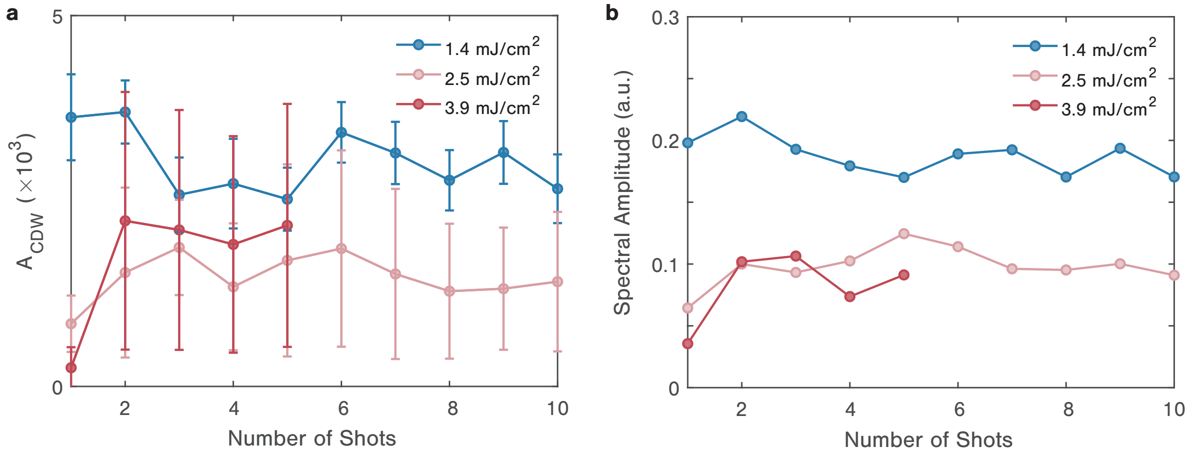

Fig. 4. Suppression of the CDW amplitude mode during switching to the H state at 7.8 K. Amplitudes derived from **a**, time-domain fits and **b**, Fourier transforms of the oscillatory response in the single-shot transient reflectivity traces in Fig. S3 with fluences of 1.4 mJ/cm^2 (blue), 2.5 mJ/cm^2 (light red) and 3.9 mJ/cm^2 (red). The suppression of the amplitude mode is strongest for the first shots at 2.5 mJ/cm^2 and 3.9 mJ/cm^2. Note that the spectral amplitudes shown in **b** correspond to the Fourier amplitude at 2.3 THz.

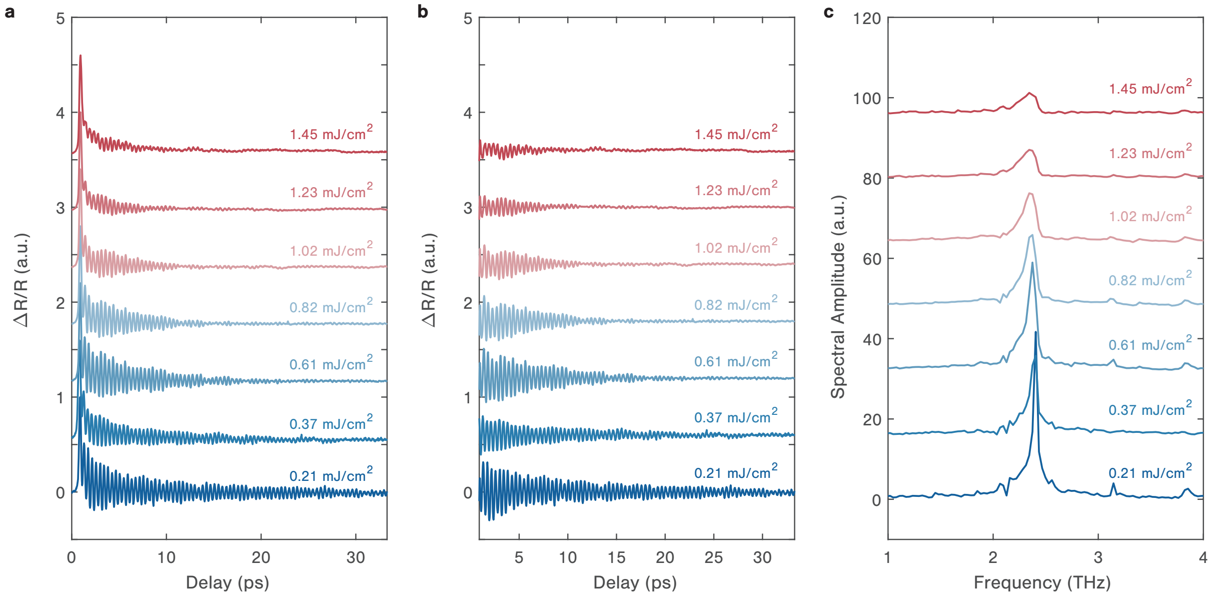

Fig. 5. Conventional pump-probe transient reflectivity collected at 80 K for different photoexcitation fluences. Pump-probe measurements were collected with the same configuration as the single-shot transient reflectivity except that the laser repetition rate was changed to 1 kHz and signals were measured with balanced photodiodes and lock-in detection. Increasing the fluence above 1.45 mJ/cm^2 led to optical sample damage. **a**, Transient reflectivity traces. **b**, Oscillatory components from data in **a** with the incoherent background removed. **c**, The corresponding Fourier transforms of the oscillations in **b**.

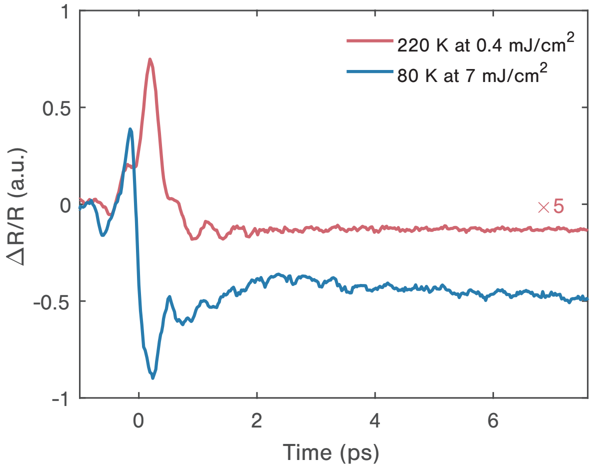

Fig. 6. Transient reflectivity traces with thermal response. Transient reflectivity traces taken at 80 K with incident fluence of 7 mJ/cm^2 (blue) and at 220 K with incident fluence of 0.4 mJ/cm^2 (red). Both traces show an initial positive reflectivity response followed by a drop in reflectivity which persists beyond the experimental time window. This steady state is attributed to a thermal response which can be readily distinguished from the non-thermal background seen at 80 K with lower fluences.

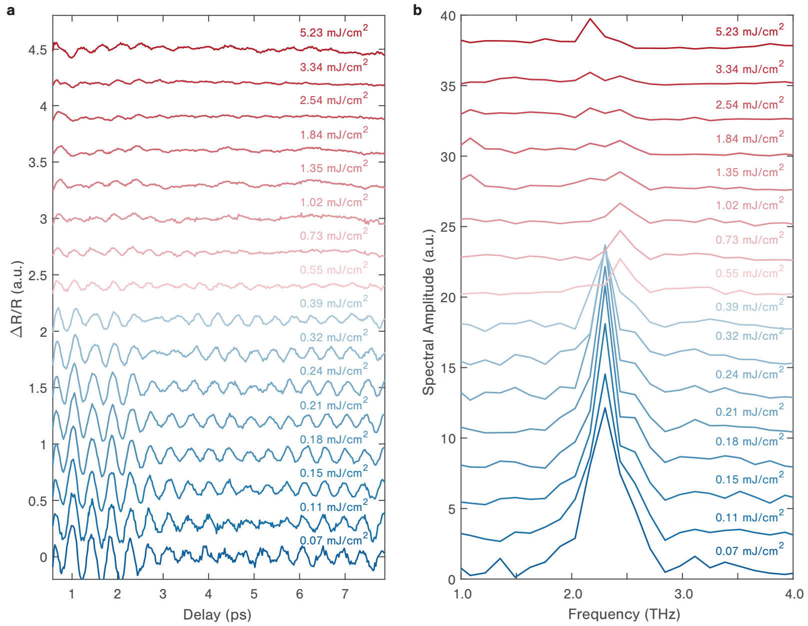

Fig. 7. Collective mode oscillations during H state formation as a function of photoexcitation fluence at 80 K. a, Oscillatory component after subtracting the incoherent background from the single-shot transient reflectivity data at 80 K with different fluences. **b**, The corresponding Fourier transforms of the data shown in **a**.

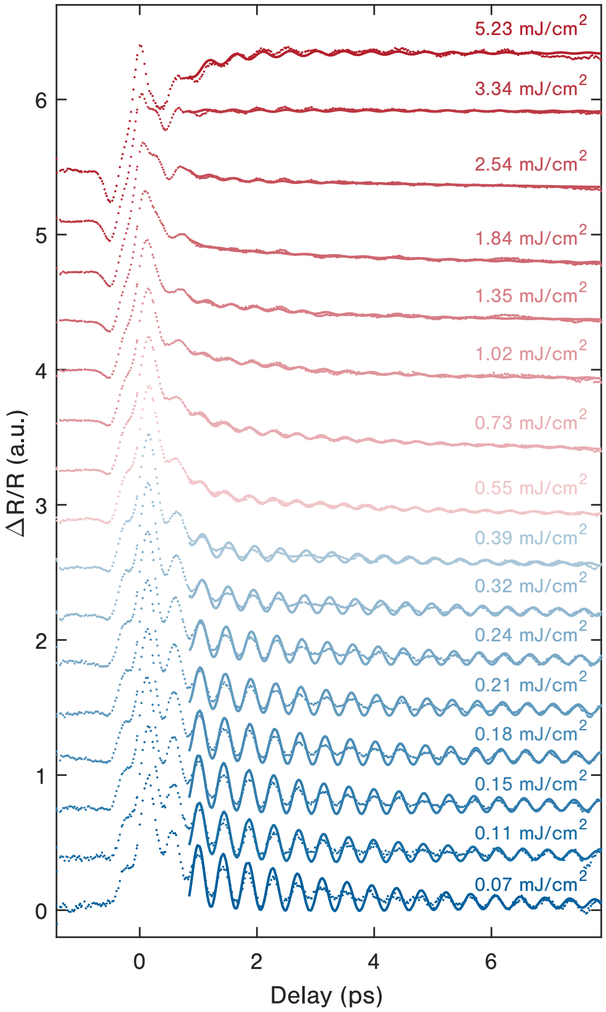

Fig. 8. Fits to the 80 K transient reflectivity signal. The 80 K transient reflectivity data from Fig. 3 (dots) is shown alongside fits (solid lines) to the model given in equation 1 of the main text. We ignore the presence of other phonon modes at low fluence and fit to a single oscillatory response. Most time-domain features are well captured by the fits.

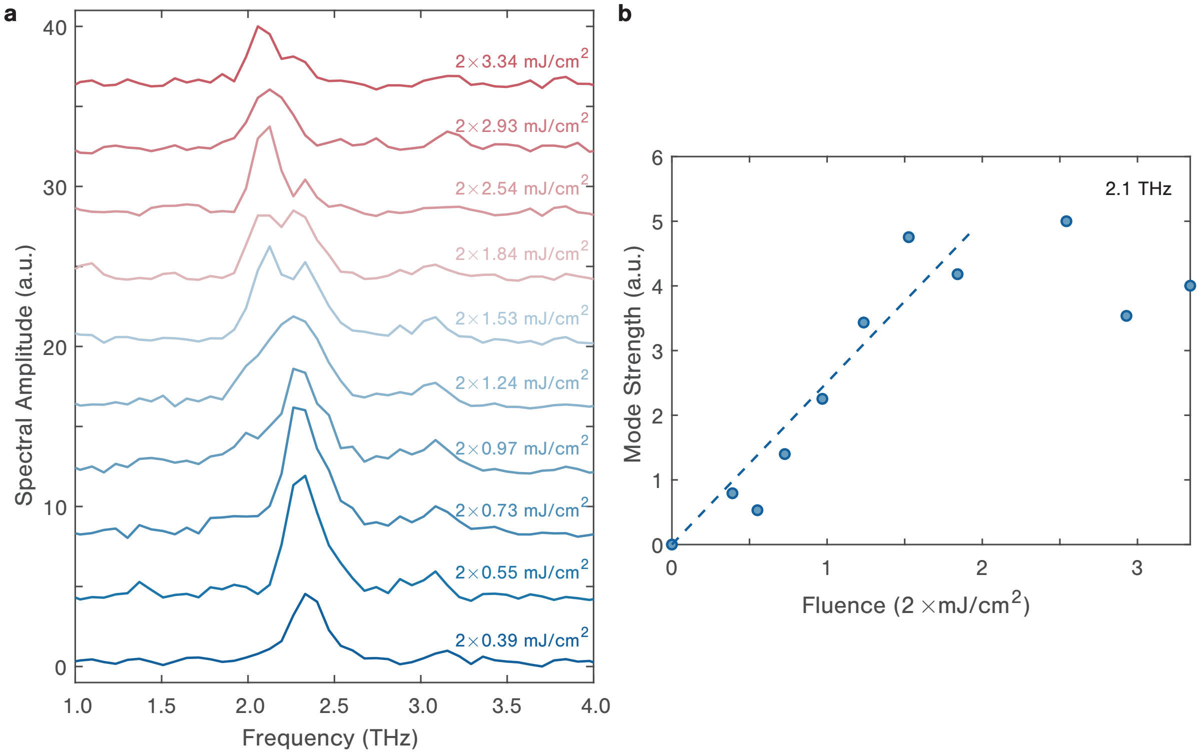

Fig. 9. Fluence dependence of the 2.1 THz mode strength. To clearly resolve the weak signal from this mode, two pump pulses with same fluence are used to cancel the amplitude mode and resolve this mode. The fluence indicated here is that of each pump pulse. **a**, Fourier transform spectra with respect to the coherent response with two pumps separated by 0.64 ps. **b**, The 2.1 THz mode strength as a function of fluence for the data in **a**. The dotted line is a guide for the eye.

F (mJ/cm^2)	N	Γ_1 (ps^{-1})	Γ_2 (ps^{-1})	Γ_{CDW} (ps^{-1})	Φ
1.4	1-10	5.0	0.33	0.10	1.9
2.5	11-20	6.0	0.27	0.10	2.3
3.9	21-25	9.0	0.05	0.10	0.7

Table 1. Fixed fit parameter values for the 7.8 K single-shot transient reflectivity data. Fixed parameter values used in the fits of the 7.8 K single-shot transient reflectivity traces in Fig. 3 and 8 with the model given in equation 2. Fixing these values was necessary to ensure consistent biexponential and oscillatory fitting across each set of data. The values were derived from the average values of unconstrained prefits of the data with the same model.

F (mJ/cm^2)	N	A_1	A_2	C	A_{CDW}	ω_0 (THz)
1.4	1	0.056	0.017	0.008	0.0036	2.3
	2	0.048	0.018	0.010	0.0037	2.3
	3	0.048	0.017	0.005	0.0026	2.4
	4	0.048	0.019	0.007	0.0027	2.3
	5	0.051	0.017	0.007	0.0025	2.3
	6	0.049	0.018	0.005	0.0034	2.3
	7	0.055	0.018	0.007	0.0031	2.3
	8	0.049	0.019	0.005	0.0028	2.3
	9	0.051	0.018	0.006	0.0032	2.3
	10	0.043	0.017	0.006	0.0027	2.4
2.5	11	0.028	0.013	0.042	0.0008	2.4
	12	0.033	0.017	0.055	0.0015	2.3
	13	0.027	0.019	0.061	0.0019	2.2
	14	0.047	0.019	0.065	0.0013	2.3
	15	0.033	0.018	0.066	0.0017	2.3
	16	0.047	0.016	0.062	0.0019	2.3
	17	0.029	0.018	0.061	0.0015	2.3
	18	0.033	0.016	0.066	0.0013	2.3
	19	0.034	0.018	0.066	0.0013	2.3
	20	0.030	0.017	0.063	0.0014	2.3
3.9	21	0.020	0.016	0.080	0.00025	2.2
	22	0.021	0.016	0.085	0.0022	2.2
	23	0.022	0.021	0.082	0.0021	2.2
	24	0.030	0.015	0.082	0.0019	2.2
	25	0.018	0.017	0.089	0.0022	2.2

Table 2. Free fit parameter values for the 7.8 K single-shot transient reflectivity data. Values obtained from fitting the 7.8 K single-shot transient reflectivity traces in Fig. 3 with the model in equation 2 having fixed the parameters given in Table 1

F (mJ/cm^2)	A_e	Γ_e (ps^{-1})	C	A_{CDW}	Γ_{CDW} (ps^{-1})	ω_0 (THz)	Φ (rad)
0.07	0.0018	0.5978	0.0003	0.5572	0.336	2.38	-0.93
0.11	0.0029	0.8418	0.0005	0.4723	0.327	2.37	-0.70
0.15	0.0034	0.7787	0.0008	0.4591	0.276	2.35	-0.87
0.18	0.0040	0.7897	0.0009	0.4404	0.281	2.34	-0.68
0.21	0.0046	0.8406	0.0010	0.4006	0.288	2.34	-0.50
0.24	0.0052	0.8871	0.0013	0.3660	0.307	2.32	-0.37
0.32	0.0068	1.1532	0.0016	0.2897	0.334	2.31	0.02
0.39	0.0084	1.2471	0.0018	0.2346	0.467	2.29	0.16
0.55	0.0124	0.8994	0.0030	0.1489	0.549	2.50	0.57
0.73	0.0153	0.6672	0.0098	0.0968	0.432	2.48	0.48
1.02	0.0144	0.6293	0.0207	0.0765	0.514	2.46	0.82
1.35	0.0120	0.6345	0.0270	0.1029	0.999	2.42	1.20
1.84	0.0114	0.5865	0.0321	0.0911	1.000	2.40	1.62
2.54	0.0072	0.3768	0.0422	0.0748	1.000	2.32	1.52
3.34	0	–	0.0497	0.0211	0.259	2.43	2.25
F (mJ/cm^2)	A_e	Γ_e (ps^{-1})	C	A_{E_g}	Γ_{E_g} (ps^{-1})	ω_0 (THz)	Φ (rad)
5.22	-0.0102	1.8434	0.0384	0.0806	0.358	2.16	0.92

Table 3. Fit parameter values for the transient reflectivity data at 80 K. Parameter values obtained from the fits of 80 K transient reflectivity to the model given by equation 5. Note that for fluences of 0.55 mJ/cm^2 and greater, the amplitude of the CDW mode approaches zero and thus the other fitted parameters do not necessarily reflect the true behavior of the CDW mode. At the highest fluence of 5.22 mJ/cm^2, the fitting function has the same form but the 2.1 THz oscillation is assigned to the E_g mode.

REFERENCES AND NOTES

1. J. Zhang, R. D. Averitt, Dynamics and control in complex transition metal oxides. *Annu. Rev. Mater. Sci.* **44**, 19–43 (2014).

2. D. N. Basov, R. D. Averitt, D. Hsieh, Towards properties on demand in quantum materials. *Nat. Mater.* **16**, 1077–1088 (2017).

3. K. A. Cremin, J. Zhang, C. C. Homes, G. D. Gu, Z. Sun, M. M. Fogler, A. J. Millis, D. N. Basov, R. D. Averitt, Photoenhanced metastable c-axis electrodynamics in stripe-ordered cuprate $La_{1.885}Ba_{0.115}CuO_4$. *Proc. Natl. Acad. Sci. U.S.A.* **116**, 19875–19879 (2019).

4. D. Fausti, R. I. Tobey, N. Dean, S. Kaiser, A. Dienst, M. C. Hoffmann, S. Pyon, T. Takayama, H. Takagi, A. Cavalleri, Light-induced superconductivity in a stripe-ordered cuprate. *Science* **331**, 189–191 (2011).

5. M. Mitrano, A. Cantaluppi, D. Nicoletti, S. Kaiser, A. Perucchi, S. Lupi, P. Di Pietro, D. Pontiroli, M. Riccò, S. R. Clark, A. Cavalleri Possible light-induced superconductivity in K_3C_{60} at high temperature. *Nature* **530**, 461–464 (2016).

6. M. Först, C. Manzoni, S. Kaiser, Y. Tomioka, Y. Tokura, R. Merlin, A. Cavalleri, Nonlinear phononics as an ultrafast route to lattice control. *Nat. Phys.* **7**, 854–856 (2011).

7. T. F. Nova, A. Cartella, A. Cantaluppi, M. Forst, D. Bossini, R. V. Mikhaylovskiy, A. V. Kimel, R. Merlin, A. Cavalleri, An effective magnetic field from optically driven phonons. *Nat. Phys.* **13**, 132–136 (2017).

8. X. Li, T. Qiu, J. Zhang, E. Baldini, J. Lu, A. M. Rappe, K. A. Nelson, Terahertz field– induced ferroelectricity in quantum paraelectric $SrTiO_3$. *Science* **364**, 1079–1082 (2019).

9. Y. H. Wang, H. Steinberg, P. Jarillo-Herrero, N. Gedik, Observation of Floquet-Bloch states on the surface of a topological insulator. *Science* **342**, 453–457 (2013).

10. F. Mahmood, C.-K. Chan, Z. Alpichshev, D. Gardner, Y. Lee, P. A. Lee, N. Gedik, Selective scattering between Floquet–Bloch and Volkov states in a topological insulator. *Nat. Phys.* **12**, 306–310 (2016).

11. L. Stojchevska, I. Vaskivskyi, T. Mertelj, P. Kusar, D. Svetin, S. Brazovskii, D. Mihailovic, Ultrafast switching to a stable hidden quantum state in an electronic crystal. *Science* **344**, 177–180 (2014).

12. J. Zhang, X. Tan, M. Liu, S. W. Teitelbaum, K. W. Post, F. Jin, K. A. Nelson, D. N. Basov, W. Wu, R. D. Averitt, Cooperative photoinduced metastable phase control in strained manganite films. *Nat. Mater.* **15**, 956–960 (2016).

13. V. R. Morrison, R. P. Chatelain, K. L. Tiwari, A. Hendaoui, A. Bruhács, M. Chaker, B. J. Siwick, A photoinduced metal-like phase of monoclinic VO_2 revealed by ultrafast electron diffraction. *Science* **346**, 445–448 (2014).

14. M. R. Otto, L. P. R. de Cotret, D. A. Valverde-Chavez, K. L. Tiwari, N. Émond, M. Chaker, D. G. Cooke, B. J. Siwick, How optical excitation controls the structure and properties of vanadium dioxide. *Proc. Natl. Acad. Sci.* **116**, 450–455 (2019).

15. X. Shi, W. You, Y. Zhang, Z. Tao, P. M. Oppeneer, X. Wu, R. Thomale, K. Rossnagel, M. Bauer, H. Kapteyn, M. Murnane, Ultrafast electron calorimetry uncovers a new long-lived metastable state in 1*T*-$TaSe_2$ mediated by mode-selective electron-phonon coupling. *Sci Adv.* **5**, eaav4449 (2019).

16. A. S. McLeod, J. Zhang, M. Q. Gu, F. Jin, G. Zhang, K. W. Post, X. G. Zhao, A. J. Millis, W. B. Wu, J. M. Rondinelli, R. D. Averitt, D. N. Basov, Multi-messenger nanoprobes of hidden magnetism in a strained manganite. *Nat. Mater.* **19**, 397–404 (2020).

17. Q. M. Liu, D. Wu, Z. A. Li, L. Y. Shi, Z. X. Wang, S. J. Zhang, T. Lin, T. C. Hu, H. F. Tian, J. Q. Li, T. Dong, N. L. Wang, Photoinduced multistage phase transitions in Ta_2NiSe_5. *Nat. Commun.* **12**, 2050 (2021).

18. S. W. Teitelbaum, T. Shin, J. W. Wolfson, Y.-H. Cheng, I. J. Porter, M. Kandyla, K. A. Nelson, Real-time observation of a coherent lattice transformation into a high-symmetry phase. *Phys. Rev. X* **8**, 031081 (2018).

19. S. W. Teitelbaum, B. K. Ofori-Okai, Y.-H. Cheng, J. Zhang, F. Jin, W. Wu, R. D. Averitt, K. A. Nelson, Dynamics of a persistent insulator-to-metal transition in strained manganite films. *Phys. Rev. Lett.* **123**, 267201 (2019).

20. T. Ritschel, J. Trinckauf, K. Koepernik, B. Büchner, M. v. Zimmermann, H. Berger, Y. I. Joe, P. Abbamonte, J. Geck, Orbital textures and charge density waves in transition metal dichalcogenides. *Nat. Phys.* **11**, 328–331 (2015).

21. Q. Stahl, M. Kusch, F. Heinsch, G. Garbarino, N. Kretzschmar, K. Hanff, K. Rossnagel, J. Geck, T. Ritschel, Collapse of layer dimerization in the photo-induced hidden state of 1*T*-TaS_2. *Nat. Commun.* **11**, 1247 (2020).

22. J. A. Wilson, F. J.. Di Salvo, S. Mahajan, Charge-density waves and superlattices in the metallic layered transition metal dichalcogenides. *Adv. Phys.* **24**, 117–201 (1975).

23. B. Sipos, A. F. Kusmartseva, A. Akrap, H. Berger, L. Forró, E. Tutiš, From Mott state to superconductivity in 1*T*-TaS_2. *Nat. Mater.* **7**, 960–965 (2008).

24. I. Vaskivskyi, J. Gospodaric, S. Brazovskii, D. Svetin, P. Sutar, E. Goreshnik, I. A. Mihailovic, T. Mertelj, D. Mihailovic, Controlling the metal-to-insulator relaxation of the metastable hidden quantum state in 1*T*-TaS_2. *Sci. Adv.* **1**, e1500168 (2015).

25. I. Vaskivskyi, I. A. Mihailovic, S. Brazovskii, J. Gospodaric, T. Mertelj, D. Svetin, P. Sutar, D. Mihailovic, Fast electronic resistance switching involving hidden charge density wave states. *Nat. Commun.* **7**, 11442 (2016).

26. J. Ravnik, M. Diego, Y. Gerasimenko, Y. Vaskivskyi, I. Vaskivskyi, T. Mertelj, J. Vodeb, D. Mihailovic, A time-domain phase diagram of metastable states in a charge ordered quantum material. *Nat. Commun.* **12**, 2323 (2021).

27. Y. A. Gerasimenko, P. Karpov, I. Vaskivskyi, S. Brazovskii, D. Mihailovic, Intertwined chiral charge orders and topological stabilization of the light-induced state of a prototypical transition metal dichalcogenide. *npj Quantum Mater.* **4**, 32 (2019).

28. K. Sun, S. Sun, C. Zhu, H. Tian, H. Yang, J. Li, Hidden CDW states and insulator-tometal transition after a pulsed femtosecond laser excitation in layered chalcogenide 1*T*-$TaS_{2-x}Se_x$. *Sci Adv.* **4**, eaas9660 (2018).

29. J. Ravnik, I. Vaskivskyi, T. Mertelj, D. Mihailovic, Real-time observation of the coherent transition to a metastable emergent state in 1*T*-TaS_2. *Phys. Rev. B* **97**, 075304 (2018).

30. T. Shin, J. W. Wolfson, S. W. Teitelbaum, M. Kandyla, K. A. Nelson, Dual echelon femtosecond single-shot spectroscopy. *Rev. Sci. Instrum.* **85**, 083115 (2014).

31. N. Dean, J. C. Petersen, D. Fausti, R. I. Tobey, S. Kaiser, L. Gasparov, H. Berger, A. Cavalleri, Polaronic conductivity in the photoinduced phase of 1*T*-TaS_2. *Phys. Rev. Lett.* **106**, 016401 (2011).

32. R. Ulbricht, E. Hendry, J. Shan, T. F. Heinz, M. Bonn, Carrier dynamics in semiconductors studied with time-resolved terahertz spectroscopy. *Rev. Mod. Phys.* **83**, 543– 586 (2011.

33. Y. A. Gerasimenko, I. Vaskivskyi, M. Litskevich, J. Ravnik, J. Vodeb, M. Diego, V. Kabanov, D. Mihailovic, Quantum jamming transition to a correlated electron glass in 1*T*-TaS_2. *Nat. Mater.* **18**, 1078–1083 (2019).

34. J. Demsar, L. Forró, H. Berger, D. Mihailovic, Femtosecond snapshots of gap-forming charge-density-wave correlations in quasi-two-dimensional dichalcogenides 1*T*-TaS_2 and 2*H*-$TaSe_2$. *Phys. Rev. B* **66**, 041101 (2002).

35. Y. Toda, K. Tateishi, S. Tanda, Anomalous coherent phonon oscillations in the commensurate phase of the quasi-two-dimensional 1T−TaS_2 compound. *Phys. Rev. B* **70**, 033106 (2004).

36. L. Perfetti, P. A. Loukakos, M. Lisowski, U. Bovensiepen, M. Wolf, H. Berger, S. Biermann, A. Georges, Femtosecond dynamics of electronic states in the Mott insulator 1T-TaS_2 by time resolved photoelectron spectroscopy. *New J. Phys.* **10**, 053019 (2008).

37. A. Mann, E. Baldini, A. Odeh, A. Magrez, H. Berger, F. Carbone, Probing the coupling between a doublon excitation and the charge-density wave in TaS_2 by ultrafast optical spectroscopy. *Phys. Rev. B* **94**, 115122 (2016).

38. E. Baldini, C. A. Belvin, M. Rodriguez-Vega, I. O. Ozel, D. Legut, A. Kozłowski, A. M. Oleś, K. Parlinski, P. Piekarz, J. Lorenzana, G. A. Fiete, N. Gedik, Discovery of the soft electronic modes of the trimeron order in magnetite. *Nat. Phys.* **16**, 541–545 (2020).

39. S. Sugai, K. Murase, S. Uchida, S. Tanaka, Comparison of the soft modes in tantalum dichalcogenides. *Phys. B+ C* **105**, 405–409 (1981).

40. M. Eichberger, H. Schäfer, M. Krumova, M. Beyer, J. Demsar, H. Berger, G. Moriena, G. Sciaini, R. J. D. Miller, Snapshots of cooperative atomic motions in the optical suppression of charge density waves. *Nature* **468**, 799–802 (2010).

41. T. N. Ikeda, H. Tsunetsugu, K. Yonemitsu, Photoinduced dynamics of commensurate charge density wave in 1*T*-TaS_2 based on three-orbital hubbard model. *Appl. Sci.* **9**, 70 (2019).

42. J. Zhang, C. Lian, M. Guan, W. Ma, H. Fu, H. Guo, S. Meng, Photoexcitation induced quantum dynamics of charge density wave and emergence of a collective mode in 1*T*-TaS_2. *Nano Lett.* **19**, 6027–6034 (2019).

43. S. M. Teo, B. K. Ofori-Okai, C. A. Werley, K. A. Nelson, Invited article: Single-shot THz detection techniques optimized for multidimensional THz spectroscopy. *Rev Sci Instrum.* **86**, 051301 (2015).

44. L. Ma, C. Ye, Y. Yu, X. F. Lu, X. Niu, S. Kim, D. Feng, D. Tománek, Y.-W. Son, X. H. Chen, Y. Zhang, A metallic mosaic phase and the origin of mott-insulating state in 1*T*-TaS_2. *Nat. Commun.* **7**, 10956 (2016).

45. C. W. Nicholson, F. Petocchi, B. Salzmann, C. Witteveen, M. Rumo, G. Kremer, F. O. von Rohr, P. Werner, C. Monney, Modified interlayer stacking and insulator to correlated-metal transition driven by uniaxial strain in *1T*-TaS_2. https://arxiv.org/abs/2204.05598.

46. Z. Sun, A. J. Millis, Transient trapping into metastable states in systems with competing orders. *Phys. Rev. X* **10**, 021028 (2020).

47. A. Zong, P. E. Dolgirev, A. Kogar, E. Ergeçen, M. B. Yilmaz, Y.-Q. Bie, T. Rohwer, I.-C. Tung, J. Straquadine, X. Wang, Y. Yang, X. Shen, R. Li, J. Yang, S. Park, M. C. Hoffmann, B. K. Ofori-Okai, M. E. Kozina, H. Wen, X. Wang, I. R. Fisher, P. Jarillo-Herrero, N. Gedik, Dynamical slowing-down in an ultrafast photoinduced phase transition. *Phys. Rev. Lett.* **123**, 097601 (2019).

48. A. Zong, A. Kogar, Y.-Q. Bie, T. Rohwer, C. Lee, E. Baldini, E. Ergeçen, M. B. Yilmaz, B. Freelon, E. J. Sie, H. Zhou, J. Straquadine, P. Walmsley, P. E. Dolgirev, A. V. Rozhkov, I. R. Fisher, P. Jarillo-Herrero, B. V. Fine, N. Gedik, Evidence for topological defects in a photoinduced phase transition. *Nat. Phys.* **15**, 27–31 (2019).

49. A. Kogar, A. Zong, P. E. Dolgirev, X. Shen, J. Straquadine, Y.-Q. Bie, X. Wang, T. Rohwer, I.-C. Tung, Y. Yang, R. Li, J. Yang, S. Weathersby, S. Park, M. E. Kozina, E. J. Sie, H. Wen, P. Jarillo-Herrero, I. R. Fisher, X. Wang, N. Gedik, Light-induced charge density wave in $LaTe_3$. *Nat. Phys.* **16**, 159–163 (2020).

50. P. E. Dolgirev, M. H. Michael, A. Zong, N. Gedik, E. Demler, Self-similar dynamics of order parameter fluctuations in pump-probe experiments. *Phys. Rev. B* **101**, 174306 (2020).

51. A. Zong, P. E. Dolgirev, A. Kogar, Y. Su, X. Shen, J. A. W. Straquadine, X. Wang, D. Luo, M. E. Kozina, A. H. Reid, R. Li, J. Yang, S. P. Weathersby, S. Park, E. J. Sie, P. Jarillo-Herrero, I. R. Fisher, X. Wang, E. Demler, N. Gedik, Role of equilibrium fluctuations in light-induced order. *Phys. Rev. Lett.* **127**, 227401 (2021).

52. P. C. Hohenberg, B. I. Halperin, Theory of dynamic critical phenomena. *Rev. Mod. Phys.* **49**, 435–479 (1977).

53. Y. Lemonik, A. Mitra, Quench dynamics of superconducting fluctuations and optical conductivity in a disordered system. *Phys. Rev. B* **98**, 214514 (2018).

54. J. W. Park, J. Lee, H. W. Yeom, Zoology of domain walls in quasi-2D correlated charge density wave of 1*T*-TaS_2. *npj Quantum Mater.* **6**, 32 (2021).

55. S.-Y. Xu, Q. Ma, Y. Gao, A. Kogar, A. Zong, A. M. M. Valdivia, T. H. Dinh, S.-M. Huang, B. Singh, C.-H. Hsu, T.-R. Chang, J. P. C. Ruff, K. Watanabe, T. Taniguchi, H. Lin, G. Karapetrov, D. Xiao, P. Jarillo-Herrero, N. Gedik, Spontaneous gyrotropic electronic order in a transition-metal dichalcogenide. *Nature* **578**, 545–549 (2020).

56. V. A. Stoica, N. Laanait, C. Dai, Z. Hong, Y. Yuan, Z. Zhang, S. Lei, M. R. McCarter, A. Yadav, A. R. Damodaran, S. Das, G. A. Stone, J. Karapetrova, D. A. Walko, X. Zhang, L. W. Martin, R. Ramesh, L.-Q. Chen, H. Wen, V. Gopalan, J. W. Freeland, Optical creation of a supercrystal with three-dimensional nanoscale periodicity. *Nat. Mater.* **18**, 377–383 (2019).

57. M. Budden, T. Gebert, M. Buzzi, G. Jotzu, E. Wang, T. Matsuyama, G. Meier, Y. Laplace, D. Pontiroli, M. Ricco, F. Schlawin, D. Jaksch, A. Cavalleri, Evidence for metastable photoinduced superconductivity in K_3C_{60}. *Nat. Phys.* **17**, 611–618 (2021).

58. R. Inada, Y. Ōnuki, S. Tanuma, Hall effect of 1*T*-TaS_2. *Phys. Lett. A* **69**, 453–456 (1979).

59. A. Zong, X. Shen, A. Kogar, L. Ye, C. Marks, D. Chowdhury, T. Rohwer, B. Freelon, S. Weathersby, R. Li, J. Yang, J. Checkelsky, X. Wang, N. Gedik, Ultrafast manipulation of mirror domain walls in a charge density wave. *Sci Adv.* **4**, eaau5501 (2018).

60. R. E. Glover III, M. Tinkham, Conductivity of superconducting films for photon energies between 0.3 and $40kT_c$. *Phys. Rev.* **108**, 243–256 (1957).

61. N. V. Smith, Classical generalization of the Drude formula for the optical conductivity. *Phys. Rev. B* **64**, 155106 (2001).

62. T. L. Cocker, D. Baillie, M. Buruma, L. V. Titova, R. D. Sydora, F. Marsiglio, F. A. Hegmann, Microscopic origin of the Drude-Smith model. *Phys. Rev. B* **96**, 205439 (2017).

63. G. M. Turner, M. C. Beard, C. A. Schmuttenmaer, Carrier localization and cooling in dye-sensitized nanocrystalline titanium dioxide. *J. Phys. Chem. B* **106**, 11716–11719 (2002).

64. D. G. Cooke, A. N. MacDonald, A. Hryciw, J. Wang, Q. Li, A. Meldrum, F. Hegmann, Transient terahertz conductivity in photoexcited silicon nanocrystal films. *Phys. Rev. B* **73**, 193311 (2006).

65. C. Richter, C. A. Schmuttenmaer, Exciton-like trap states limit electron mobility in TiO_2 nanotubes. *Nat. Nanotechnol.* **5**, 769–772 (2010).

66. R. E. Schwall, G. R. Stewart, T. H. Geballe, Low-temperature specific heat of layered compounds. *J. Low Temp. Phys.* **22**, 557–567 (1976).

67. A. R. Beal, H. P. Hughes, W. Y. Liang, The reflectivity spectra of some group Va transition metal dichalcogenides. *J. Phys. C: Solid State Phys.* **8**, 4236 (1975).

68. H. J. Zeiger, J. Vidal, T. K. Cheng, E. P. Ippen, G. Dresselhaus, M. S. Dresselhaus, Theory for displacive excitation of coherent phonons. *Phys. Rev. B* **45**, 768–778 (1992).

69. T. Stevens, J. Kuhl, R. Merlin, Coherent phonon generation and the two stimulated raman tensors. *Phys. Rev. B* **65**, 144304 (2002).
